# Artificial Intelligence‐Based Multimodal Prediction of Postoperative Adjuvant Immunotherapy Benefit in Urothelial Carcinoma: Results From the Phase III, Multicenter, Randomized, IMvigor010 Trial

**DOI:** 10.1002/mco2.70324

**Published:** 2025-08-25

**Authors:** Xiatong Huang, Wenjun Qiu, Yuyun Kong, Qiyun Ou, Qianqian Mao, Yiran Fang, Zhouyang Fan, Jiani Wu, Xiansheng Lu, Wenchao Gu, Peng Luo, Junfen Wang, Jianping Bin, Yulin Liao, Min Shi, Zuqiang Wu, Huiying Sun, Yunfang Yu, Wangjun Liao, Dongqiang Zeng

**Affiliations:** ^1^ Department of Oncology Nanfang Hospital Southern Medical University Guangzhou Guangdong China; ^2^ Cancer Center the Sixth Affiliated Hospital School of Medicine South China University of Technology Foshan China; ^3^ Foshan Key Laboratory of Translational Medicine in Oncology The Sixth Affiliated Hospital School of Medicine South China University of Technology Foshan China; ^4^ Guangdong Provincial Key Laboratory of Malignant Tumor Epigenetics and Gene Regulation Department of Ultrasound Sun Yat‐sen Memorial Hospital Sun Yat‐sen University Guangzhou China; ^5^ The First School of Clinical Medicine Southern Medical University Guangzhou Guangdong China; ^6^ Department of Pathology Nanfang Hospital Southern Medical University Guangzhou Guangdong China; ^7^ Department of Diagnostic and Interventional Radiology University of Tsukuba Ibaraki Japan; ^8^ Department of Oncology Zhujiang Hospital Southern Medical University Guangzhou Guangdong China; ^9^ Department of Gastroenterology Nanfang Hospital Southern Medical University Guangzhou China; ^10^ Department of Cardiology Nanfang Hospital Southern Medical University Guangzhou Guangdong China; ^11^ Guangdong Provincial Key Laboratory of Malignant Tumor Epigenetics and Gene Regulation Guangdong‐Hong Kong Joint Laboratory for RNA Medicine Department of Medical Oncology Sun Yat‐sen Memorial Hospital Sun Yat‐sen University Guangzhou China; ^12^ Guangdong Provincial Key Laboratory of Cancer Pathogenesis and Precision Diagnosis and Treatment AI and joint Big Data Laboratory Department of Medical Oncology Sun Yat‐sen Memorial Hospital Sun Yat‐sen University Shanwei China; ^13^ Department of Breast Surgery The First Affiliated Hospital Jinan University Guangzhou China; ^14^ Institute for AI in Medicine and faculty of Medicine Macau University of Science and Technology Taipa Macao China

**Keywords:** adjuvant immunotherapy, biomarker, decision tree model, predictive score, urothelial carcinoma

## Abstract

While circulating tumor DNA (ctDNA) testing has demonstrated utility in identifying muscle‐invasive urothelial carcinoma (MIUC) patients likely to benefit from adjuvant immunotherapy, the prognostic value of transcriptome data from surgical specimens remains underexplored. Using transcriptomic and ctDNA data from the IMvigor010 trial, we developed an artificial intelligence (AI)‐driven biomarker to predict immunotherapy response in urothelial carcinoma, termed UAIscore. Patients with high UAIscore had significantly better outcomes in the atezolizumab arm versus the observation arm. Notably, the predictive performance of the UAIscore consistently outperformed that of ctDNA, tTMB, and PD‐L1, highlighting its value as an independent biomarker. Moreover, combining ctDNA, tTMB, and PD‐L1 with the UAIscore further improved predictive accuracy, underscoring the importance of integrating multi‐modality biomarkers. Further analysis of molecular subtypes revealed that the luminal subtype tends to be sensitive to adjuvant immunotherapy, as it may exhibit the highest level of immune infiltration and the lowest degree of hypoxia. Remarkably, we elucidated the role of the NF‐κB and TNF‐α pathways in mediating immunotherapy resistance within the immune‐enriched tumor microenvironment. These findings stratify patients likely to respond to adjuvant immunotherapy, concurrently providing a mechanistic rationale for combination therapies to augment immunotherapy efficacy in urothelial carcinoma.

## Graphical abstract

Based on transcriptome and ctDNA data from the IMvigor010 clinical trial, we sought to identify biomarkers that can predict outcomes following adjuvant immunotherapy for urothelial carcinoma by means of artificial intelligence, which is termed as UAIscore. Combining ctDNA, tTMB, and PD‐L1 with the UAIscore, as multi‐modality biomarkers, further improved predictive accuracy.

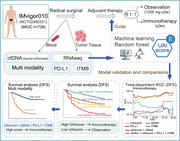

## 1 Introduction

Primary urothelial carcinomas include bladder and upper urothelial carcinomas, of which approximately 25% are muscle‐invasive urothelial carcinomas (MIUCs) [1, 2]. The current standard of treatment is radical resection of the bladder or renal ureter [1, 2]. However, MIUC has a high risk of recurrence, with approximately half of the patients experiencing local or distal recurrence or metastasis within 2 years of radical resection and 5‐year survival rates of 60% or less [1, 3]. In recent years, significant breakthroughs have been made in the application of immune checkpoint blockades (ICBs) in urothelial carcinoma. The Food and Drug Administration (FDA) has approved a variety of ICBs for the treatment of advanced urothelial carcinoma, including nivolumab, avelumab, and pembrolizumab [3, 4, 5]. Nevertheless, the promotion of adjuvant immunotherapy has encountered many challenges. At present, only CheckMate274 [3] clinical study has been successful, and nivolumab is the only drug recommended for adjuvant treatment after radical resection of urothelial carcinoma. At a median follow‐up of about 20 months, 48.2% of patients in the nivolumab group and 57.3% of patients in the placebo group had relapsed or died. We observed that in another large randomized clinical study (IMvigor010) [6], the ICB (anti‐PD‐L1) treatment group was no better than the observation group. IMvigor010 (NCT02450331), the largest and first completed Phase III randomized clinical trial of adjuvant therapy for MIUC, compared the results of the atezolizumab treatment group with the observation group. Unfortunately, the study did not meet its primary study endpoint [6, 7].

Reflecting on the failure of IMvigor010, we realized that finding biomarkers that can predict the efficacy of ICBs and thus screen patients who will respond to ICBs was crucial for accurately improving the prognosis of urothelial carcinoma in recent years. To date, the biomarkers most commonly used to predict the efficacy of ICBs include expression level of programmed cell death‐ligand 1 (PD‐L1) [8], tumor mutational burden (TMB) [9], microsatellite instability (MSI) [10], and circulating tumor DNA (ctDNA) [7]. Subsequent exploratory biomarker analysis of IMvigor010 also revealed the predictive ability of ctDNA for ICBs [7]. Indeed, ctDNA as a biomarker still has limitations, such as the high cost of ctDNA testing, false‐positive results, and no treatment recommendation for ctDNA‐negative patients.

With the rapid development of artificial intelligence (AI) and multi‐omics, their potential in the medical field has become increasingly evident [11, 12, 13]. Machine learning and deep learning techniques have been widely applied in various studies related to cancer diagnosis, prognostic prediction, treatment planning, and so on [11, 12, 14, 15]. Among these, the random forest classifier is one of the popular models. For urothelial carcinoma, various molecular subtypes have been constructed based on genome, transcriptome, epigenome, and multi‐omics combinations, including transcriptional component types that predict the response to neoadjuvant chemotherapy [14], genomic subtypes that predict the efficacy of ICBs in advanced urothelial carcinoma [15], and spatial immunophenotype predicting the efficacy of ICBs in urothelial cancers with distant metastases that do not match the primary site [16]. However, there are currently no widely available clinical biomarkers for MIUC to predict the response to adjuvant immunotherapy.

In this study, based on the transcriptomic data from IMvigor010 cohort (*n* = 728 high‐risk MIUC), we constructed a predictive score for survival benefit from adjuvant immunotherapy in urothelial carcinoma (UAIscore), using machine learning algorithms. [17, 18, 19, 20] The UAIscore could be used as an independent biomarker and combined with ctDNA as a multi‐modality, which could accurately select patients who are likely to respond to immunotherapy in urothelial carcinoma. Importantly, a variety of influencing factors were comprehensively identified, including the infiltration of tumor immune microenvironment and clinical characteristics such as relapse status as well as tumor stage. In patients who did not respond, drug resistance factors were more common such as hypoxia, angiogenesis, insulin‐like growth factor 1 (IGF‐1), and fibroblast growth factor receptor (FGFR) in accordance with previous studies [21, 22, 23, 24]. Interestingly, a few patients did not show differences in prognosis based on variations in the degree of immune infiltration, so it was necessary to uncover the mechanism of other important factors such as nuclear factor kappa‐light‐chain‐enhancer of activated B cells (NF‐κB) and tumor necrosis factor alpha (TNF‐α) pathways leading to resistance in immune‐enriched tumor microenvironment (TME) [25, 26]. Thus, we have initially revealed the possible mechanisms of immune benefit or resistance, providing a preliminary analytical template and potential clinical basis for the precise stratification of immunotherapy and the development of novel combination strategies in urothelial carcinoma.

## 2 Results

### 2.1 The Procedure of the UAIscore Conducted by the Random Forest Algorithm

**FIGURE 1:**
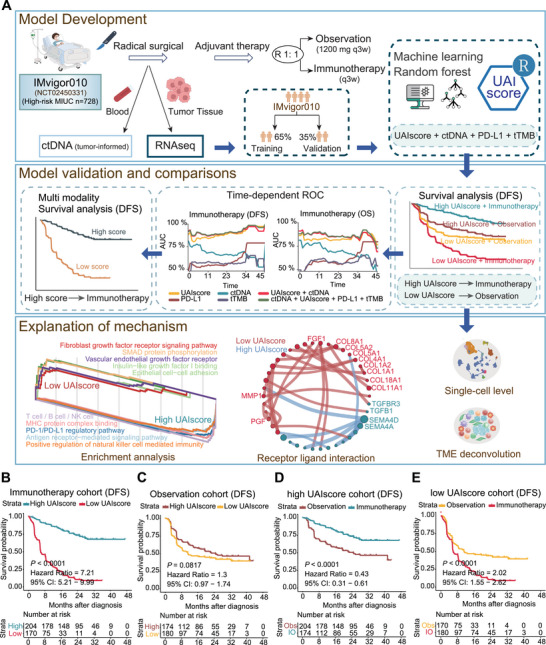
Patients with high UAIscore were more likely to benefit from immunotherapy. (A) The flowchart showed the construction of the UAIscore model and subsequent exploration of its underlying mechanism. Created by BioRender and Vecteezy. (B and C) Kaplan–Meier curves of UAIscore were generated based on DFS survival analysis in immunotherapy (B) and observation (C) cohorts of IMvigor010. (D and E) Kaplan–Meier curves of treatment were generated based on DFS survival analysis in high UAIscore (D) and low UAIscore (E) groups of IMvigor010. DFS, disease‐free survival; OS, overall survival; high, high UAIscore; low, low UAIscore; IO, immunotherapy; Obs, observation; ctDNA, ctDNA tested before treatment, tTMB, tumor mutation burden; PD‐L1, programmed cell death‐ligand 1.

We utilized data from the IMvigor010 clinical trial to construct the UAIscore for predicting survival benefit from adjuvant immunotherapy, further demonstrating the independent predictive efficacy of UAIscore and the value of multi‐modality integration. Additionally, we explored the mechanisms of immunotherapy resistance from the perspectives of TME, receptor‐ligand interactions, and single‐cell interactions, providing a foundation for combined treatment strategies (Figure 1A).

Firstly, the transcriptome data of 728 MIUC patients in the IMvigor010 clinical trial were analyzed, with the patients randomly divided into a training group (*n* = 475) and a validation group (*n* = 253) in a 6.5:3.5 ratio. Batch survival analysis was performed for disease‐free survival (DFS) in the training group, and the genes not associated with DFS (*p* > 0.05) were filtered out. Subgroup survival analysis was performed to select interaction genes whose expression levels caused survival differences in only one treatment group using the Publish R package, resulting in the identification of 91 genes. Then, random forest machine learning was employed to construct a model with 91 survival‐related marker genes to predict the prognosis of adjuvant immunotherapy, named UAIscore (details of the model construction were presented in Section 6 and Figure S1). Demographics of patients were compared, with no statistically significant differences between high and low UAIscore populations in sex and phenotype (Tables S1 and S2). Patients were stratified into high and low UAIscore groups according to the median UAIscore in the training set. Combining UAIscore stratification and treatment, all patients were divided into four groups: high UAIscore + immunotherapy (*n* = 204), high UAIscore + observation (*n* = 174), low UAIscore + immunotherapy (*n* = 170), low UAIscore + observation (*n* = 180).

### 2.2 Patients With High UAIscore Were More Likely to Benefit From Immunotherapy

Firstly, we evaluated whether UAIscore was associated with prognosis. The results showed that patients with the high UAIscore presented prolonged DFS (hazard ratio [HR] 2.85, 95% CI: 2.3–3.52, *p* < 0.0001) and OS (HR 2.88, 95% CI: 2.15–3.85, *p* < 0.0001) in IMvigor010 clinical trial (Figure S2A,B). Consistently, the high UAIscore group had significantly better outcomes (HR 1.64, 95% CI: 1.15–2.33, *p* = 0.0061, Figure S2C). Thus, the UAIscore could serve as an effective prognostic indicator.

We further examined whether the UAIscore had predictive value with respect to adjuvant immunotherapy, so that the survival analysis was conducted in the training set and the results were verified in the validation set and all patients of IMvigor010 cohort (Figure 1B–E, Figure S2D–I). Notably, among patients treated with immunotherapy, the high UAIscore group had notably longer DFS (HR 7.21, 95% CI: 5.21–9.99, *p* < 0.0001, Figure 1A) and OS (HR 6.41, 95% CI: 4.04–10.19, *p* < 0.0001, Figure S2I). No difference in DFS or OS between UAIscore arms was noted for patients who were keeping observation (Figure 1C and Figure S2I).

When considering the UAIscore for treatment selection, patients who were high UAIscore had improved DFS and overall survival in the atezolizumab arm versus the observation arm (DFS HR 0.39, 95% CI: 0.31–0.61, *p* < 0.0001, Figure 1D; OS HR 0.39, 95% CI: 0.23–0.64, *p* = 0.0002; Figure S2I). In contrast, patients with low UAIscore had favorable DFS (HR 2.02, 95% CI: 1.55–2.62, *p* < 0.0001, Figure 1E) and OS (HR 1.7, 95% CI: 1.22–2.38, *p* = 0.0019, Figure S2I) when keep observing. Therefore, as the UAIscore could accurately stratify patients, those with high UAIscore were more likely to benefit from immunotherapy, while those with low UAIscore were advised to remain under observation.

### 2.3 Temporal Analysis Revealed Enhanced Predictive Performance With Multi‐Modality

**FIGURE 2:**
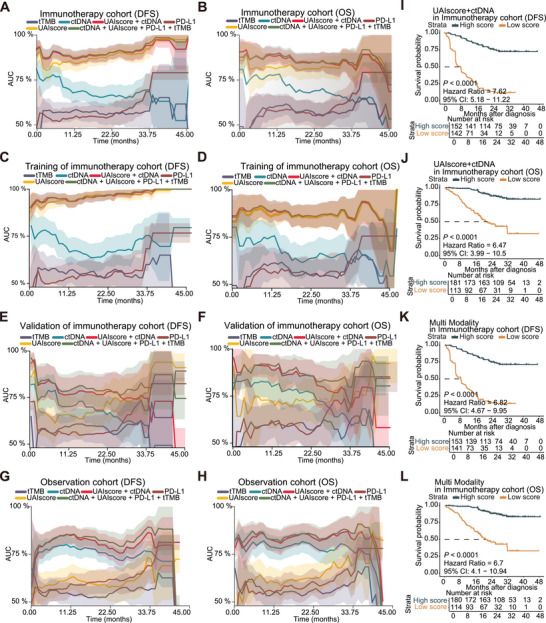
Temporal analysis revealed enhanced predictive performance with multi‐modality. (A–F) The time‐dependent AUC curve was utilized to evaluate the predictive efficacy with 95% confidence intervals of UAIscore, ctDNA, and the combination of both on DFS and OS in the immunotherapy cohorts. (G–H) The time‐dependent AUC curve was utilized to evaluate the predictive efficacy with 95% confidence intervals of UAIscore, ctDNA, and the combination of both on DFS and OS in the observation cohorts. (I–J) Kaplan–Meier curves of score obtained from the combination of UAIscore and ctDNA based on DFS and OS survival analysis in immunotherapy cohort. (K–L) Kaplan–Meier curves of multi‐modality gain from the combination of UAIscore, ctDNA, tTMB, and PD‐L1 based on DFS and OS survival analysis in immunotherapy cohort. DFS, disease‐free survival; OS, overall survival; high, high UAIscore; low, low UAIscore; IO, immunotherapy; Obs, observation; ctDNA, ctDNA tested before treatment, tTMB, tumor mutation burden; PD‐L1, programmed cell death‐ligand 1.

Currently, the biomarkers most commonly used to predict the efficacy of ICBs include expression level of PD‐L1 [8], tTMB [9], and ctDNA [7]. Additionally, the importance of multi‐omics combined biomarkers was emphasized. Therefore, the efficacy of different measures in predicting outcomes was assessed in both the immunotherapy and observation cohorts, and further evaluation was conducted to determine whether combining multiple biomarkers could enhance predictive performance. To achieve a comprehensive understanding of how the area under the curve (AUC) of the receiver operating characteristic (ROC) varies across different time points, we analyzed the time‐dependent AUC. We found that the UAIscore consistently demonstrated better predictive capability than ctDNA, tTMB, and PD‐L1 in the immunotherapy cohort (Figure 2A–F). Moreover, the combined use of ctDNA and UAIscore showed superior predictive ability compared to UAIscore alone during the initial 24 months for DFS, with a similar trend observed for OS during the initial 30 months (Figure 2A,B). In the observation cohort, although the UAIscore alone did not perform satisfactorily, its integration with ctDNA significantly enhanced the prognostic value (Figure 2G–H). Furthermore, the integration of ctDNA, tTMB, PD‐L1, and UAIscore improved the predictive accuracy in the later stages of immunotherapy survival, consistently serving as the best prognostic marker in the observation group (Figure 2G,H). The inclusion of PD‐L1 and tTMB did not markedly enhance the predictive performance of the ctDNA combined with UAIscore consistently, which might be partly due to the fact that the PD‐L1 and tTMB data obtained were binary, affecting the construction of the multi‐modality model. Consequently, enhancing the effectiveness of a multi‐omics model should involve selecting appropriate biomarkers for specific time periods.

To more clearly observe the values of AUC at specific time points, we plotted the ROC curves for 1, 2, and 3 years. The results showed that UAIscore alone exhibited superior predictive performance for DFS in the immunotherapy cohort (UAIscore 1/2/3 year AUC: 0.87/0.91/0.96; Figure S2J–L). In addition, we conducted survival analyses on the results of the combined indicators to determine whether there was a stratification effect. We found that both the combination of ctDNA and UAIscore, as well as the multi‐modality, can accurately identify patients who respond to immunotherapy (Figure 2I–L). These results demonstrated that the UAIscore could serve as an excellent and independent biomarker, and its predictive capability could be further enhanced through combination with ctDNA, tTMB, and PD‐L1 as a multi‐omic model for immunotherapy in urothelial carcinoma.

### 2.4 UAIscore Was an Independent Predictive Biomarker for Adjuvant Immunotherapy

**FIGURE 3:**
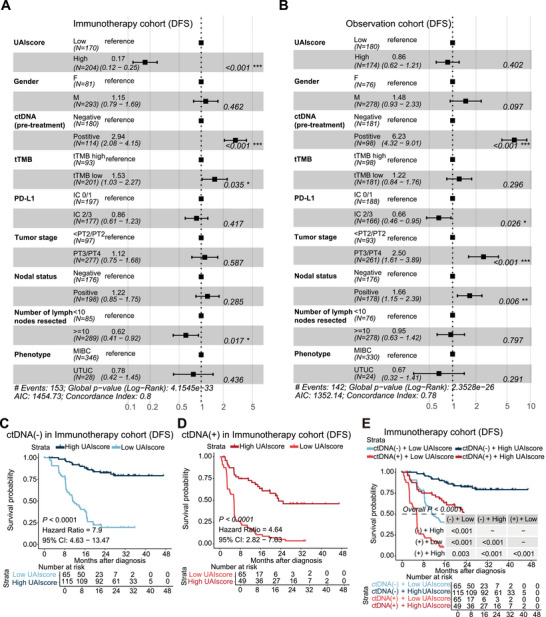
UAIscore was an independent predictive biomarker for adjuvant immunotherapy. (A) Multivariate analysis of the immunotherapy cohorts. (B) Multivariate analysis of the observation cohorts. (C and D) Kaplan–Meier curves of UAIscore in ctDNA negative and ctDNA positive groups based on DFS survival analysis in immunotherapy cohort. (E) Kaplan–Meier curves of combine of UAIscore and ctDNA based on DFS survival analysis in immunotherapy cohort. *p < 0.05; **p < 0.01; ***p < 0.001. ctDNA, ctDNA tested before treatment, tTMB, tumor mutation burden; PD‐L1, programmed cell death‐ligand 1.

To determine whether UAIscore could serve as an independent predictive biomarker, multivariate analysis was conducted separately on the immunotherapy and observation cohorts. Stratification by UAIscore showed a statistically significant difference in survival and indicated that it could serve as an independent biomarker for predicting the efficacy of immunotherapy (Figure 3A). However, stratification by UAIscore did not reveal any survival differences in the observational cohort (Figure 3B). Meanwhile, it was proved that ctDNA is a robust prognostic biomarker regardless of the treatment (Figure 3A,B). We then divided the immunotherapy cohort into ctDNA positive and ctDNA negative groups and conducted a multivariate analysis again. The results presented that UAIscore had predictive value in both groups, combined with the previous ROC analysis results, which suggested that the predictive performance can be further improved through a combination of multiple indicators (Figure S3A,B).

No survival difference was observed among ctDNA‐negative patients regardless of whether they received immunotherapy in the IMvigor010 cohort [7]. Consequently, survival analysis was conducted within the immunotherapy cohort after stratifying by ctDNA. It was found that patients with high UAIscore had the best prognosis in both groups, indicating that UAIscore could provide further guidance for treatment in ctDNA‐negative patients (Figure 3C,D). Combined analysis of UAIscore and ctDNA revealed that patients with high UAIscore and ctDNA‐negative status had the best therapeutic outcomes, suggesting that the combination of these biomarkers could more accurately identify patients who tended to be sensitive to immunotherapy (Figure 3E). These findings indicated that the UAIscore has partially compensated for the predictive limitations of ctDNA.

### 2.5 Clinical Characteristics and Molecular Subtypes and Their Potential Links to Immunotherapy Efficacy

**FIGURE 4:**
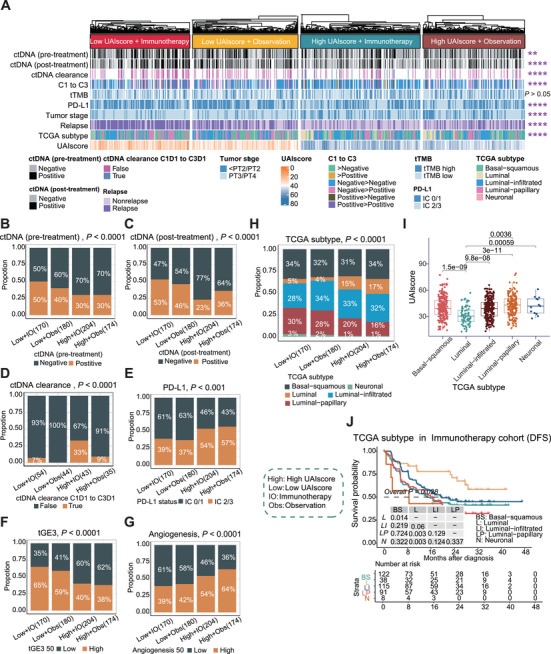
Clinical characteristics and molecular subtypes and their potential linked to immunotherapy efficacy. (A) The heatmap illustrated the distribution of each biomarker in the four groups, which are defined based on the combination of UAIscore and treatment. The biomarkers include ctDNA tested pretherapy, ctDNA tested 6 weeks after randomization (post‐treatment), ctDNA clearance at two time points, ctDNA changes at two time points (C1–C3), tTMB, PD‐L1, recurrence status, and TCGA subtype. The evaluation of these biomarkers was performed using a chi‐square test. The statistical analysis was conducted using a chi‐square test. (B–H) The bar plot provided the specific percentage of important markers that show significant differences among the four groups. These markers include ctDNA tested pretherapy, ctDNA tested post‐treatment, ctDNA clearance, PD‐L1, tGE3 50, angiogenesis 50, and TCGA subtype. The statistical analysis was conducted using chi‐square test. (I) The distribution of UAIscore among different TCGA subtypes was illustrated using box plots. The Wilcoxon test was conducted to analyze the differences in UAIscore between the two subtypes. (J) Kaplan–Meier curves were generated to perform survival analysis based on DFS for the TCGA subtype in both the immunotherapy cohorts. *p < 0.05; **p < 0.01; ***p < 0.001; ****p < 0.0001. tTMB, tumor mutation burden; PD‐L1, programmed cell death‐ligand 1; high, high UAIscore; low, low UAIscore; IO, immunotherapy; Obs, observation; DFS, disease‐free survival.

We conducted further investigations to explore clinical characteristics (Table S2). The proportion of patients who were positive for ctDNA or increased clearance of ctDNA was higher in the high UAIscore group (Figure 4A–D). Consistent with previous reports, IC 2/3 PD‐L1 [27, 28] (*p* < 0.001) and high tGE3 (CD274, IFNγ, and CXCL9) signature [29] (*p* < 0.0001) positively correlated with OS were more prevalent in the high UAIscore group, while high angiogenesis [30] (*p* < 0.0001) negatively correlated with OS was more common in the low UAIscore group (Figure 4A,E–G). Previous studies have indicated that positive for ctDNA was associated with higher recurrence rates and shorter survival [7], consistent with the finding in our study that patients in the low UAIscore group were more likely to relapse (Figure S4A,B). Interestingly, even among immunotherapy patients, those who were negative for ctDNA and were previously considered less prone to relapse had an increased likelihood of relapse if their UAIscore was low (Figure S4A,B). These findings suggested that the UAIscore may help identify ctDNA‐negative patients who were at a higher risk of relapse and ctDNA‐positive patients who may be more sensitive to immunotherapy.

We further investigated whether there were significant differences in UAIscore between different subtypes (Figure 4I and Figure S4C–E). We focused on the TCGA subtype [31] and observed that the luminal subtype was primarily distributed in the high UAIscore group, while the neuronal subtype was more prevalent in the low UAIscore group (Figure 4A,H). In the IMvigor010 cohort, survival analysis showed that the luminal subtype had the best outcomes, while the neuronal subtype had the worst survival, indicating that the TCGA subtype had prognostic values (Figure S4F–G). Additionally, survival differences among TCGA subtype were also observed in the immunotherapy cohort, suggesting that the luminal subtype was more likely to respond to immunotherapy compared to other subtypes (Figure 4J and Figure S4H). Further analysis of TME revealed that the luminal subtype displayed the highest level of immune infiltration and the lowest degree of hypoxia, which may be reasons for the favorable prognosis and the benefits from immunotherapy (Figure S4I,J). These data indicated that molecular subgroup analysis of baseline tissues may help better predict patient outcomes.

### 2.6 Molecular Characteristics and Tumor Immune Microenvironment in Patients Stratified by UAIscore

**FIGURE 5:**
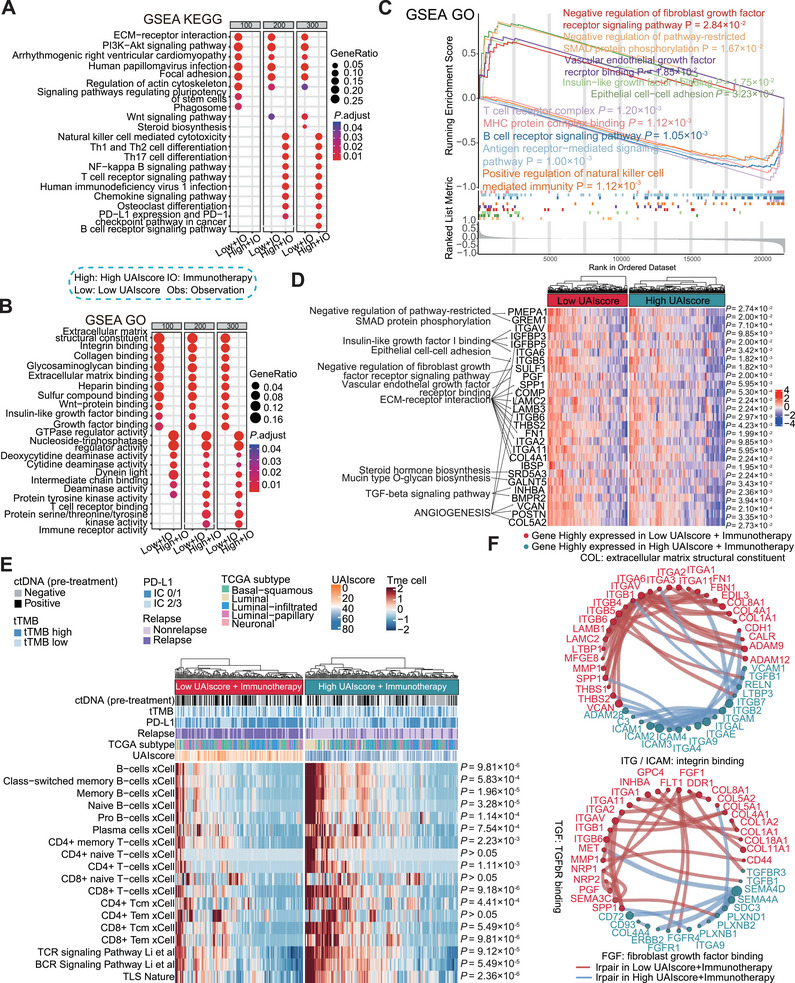
Multi‐dimensional profiling of immunotherapy response and resistance mechanisms. (A and B) From the differentially expressed genes in the high and low UAIscore groups of the immunotherapy cohort, the top 100, 200, and 300 genes in each group were selected for KEGG pathway enrichment analysis (A) and GO enrichment analysis (B). Representative pathways were chosen among those with adjusted *p* values < 0.05. (C) In the immunotherapy cohort, genes were ranked by the relative expression between the two groups, and gene set enrichment analysis (GSEA) was performed based on Gene Ontology (GO). Representative pathways were selected from those with *p*‐value < 0.05. (D) Within the representative pathways identified through GSEA enrichment analysis in the low + IO group, the expression differences of core enrichment genes were analysed using the Wilcoxon test. Genes with an adjusted *p*‐value < 0.05 were visualized in a heatmap. (E) The tumor immune microenvironment of patients in the immunotherapy cohort was analysed using the tumor microenvironment signature from the IOBR package and xCell tumor immune invasion analysis tool. Gene differential analysis was performed between the high and low UAIscore groups using the Wilcoxon test, and the distribution of immune microenvironment characteristics in the two groups was presented through heatmap. (F) The association strength of 867 ligand–receptor pairs was quantified in immunotherapy patients using the Easier package. Gene differences between groups were analyzed with the Wilcoxon test, and ligand–receptor pairs with stronger associations in each group (adjusted *p* value < 0.05) were selected and visualized in a ring chart. GSEA, gene set enrichment analysis; GO, Gene Ontology; KEGG, Kyoto Encyclopedia of Genes and Genomes; *p*, *p* value (in C); *p*, adjust *p* value (in A, B, and D–F); high, high UAIscore; low, low UAIscore; IO, immunotherapy; Obs, observation.

We wondered why patients with high UAIscore were more likely to achieve improved survival outcomes with immunotherapy, so further exploration of the underlying biology was needed. For the high UAIscore group in immunotherapy cohort, enrichment analysis revealed its potential association with various immune cells and enzymes involved in the tumor immune microenvironment (Figure S5A and Figure 5A,B). Coincidentally, gene set enrichment analysis (GSEA) also suggested that the high UAIscore group exhibited a higher enrichment of immune‐related pathways, including diverse immune cells and the PD‐1/PD‐L1 regulatory pathway (Figure 5C and Figure S5B). In contrast, the pathways enriched in the low UAIscore group were more likely characterized by the extracellular matrix and reported drug resistance factors (Figure 5C,D and Figure S5B). For instance, IGF‐1, vascular endothelial growth factor receptor (VEGFR), FGFR, and angiogenesis have been reported to be associated with tumor progression and the effect of immunotherapy [32, 33, 34]. Moreover, we utilized TME analysis and found that B cells, T cells, and tertiary lymphoid structures (TLSs) were significantly enriched in the high UAIscore group as expected, indicating a high degree of immune infiltration (Figure 5E). With a few exceptions, the existence of TLSs in tumors is generally associated with improved prognosis and clinical outcomes following immunotherapy [35, 36].

The interaction between ligands and receptors (LR) regulates cell–cell (CC) communication to modulate tumor characteristics and antitumor immune responses [37]. Thus, we quantified the strength of association for 867 ligand–receptor pairs using the Easier package and conducted a differential analysis (*p* < 0.05) (Figure 5F). Consistent with previous results, the collagen family associated with the extracellular matrix structure [38] and the fibroblast growth factor (FGF) family involved in FGF were primarily concentrated in the low UAIscore. Furthermore, most of the stronger associations in the low UAIscore exhibited HR > 1 by survival analysis (Figure S5C,D). To counteract immunotherapy resistance, novel combination strategies to change tumor immune microenvironment or target specific target ligands and receptors could be considered.

### 2.7 The TNF‐α/NF‐κB Signaling Was Associated With Immunotherapy Resistance in Immune‐Enriched TME

Unexpectedly, in the immunotherapy cohort, some patients still had high immune infiltration in the high UAIscore, while a few patients had high immune infiltration in the low UAIscore (Figure S6A). Thus, the immunotherapy cohort was further divided into four categories, including high‐UAIscore immune‐enriched TME (H‐IE), low‐UAIscore immune‐enriched TME (L‐IE), high‐UAIscore immune‐desert TME (H‐ID), and low‐UAIscore immune‐desert TME (L‐ID). Interestingly, patients within the same UAIscore group did not show differences in prognosis based on variations in the degree of immune infiltration (Figure S6B,C). The H‐IE group exhibited high PD‐L1 expression and substantial T‐cell infiltration (Figures S6A and S7A), characteristics that typically predict a positive response to anti‐PD‐L1 therapy, yet did not initially respond, which was defined as primary drug resistance in previous literature [39]. These contradictions created challenges for only TME stratification instructing immunotherapy, so that other important factors influencing the response to immunotherapy must be uncovered.

We then focused on the H‐IE and L‐IE groups to investigate the reasons for the different responses to immunotherapy despite similar immune microenvironments. At first, we compared the differences in several clinical indicators between the two groups and found that the recurrence rate in the H‐IE group was significantly higher than that in the L‐IE group (Figures S6D and S7B–G), which may be one of the primary reasons for the failure of immunotherapy. In addition, we examined the activation status of 14 key pathways and found that the NF‐κB pathway and the TNF‐α pathway were significantly activated in the H‐IE group (Figures S6E and S7H). Previous studies have indicated that NF‐κB is a transcription factor and TNF‐α is a cytokine produced by immune cells, with an inter‐regulatory effect between the TNF‐α pathway and the NF‐κB pathway [25, 26, 40]. Both TNF‐α and NF‐κB have a bidirectional effect on the shaping of the TME and the occurrence and metastasis of tumors [41, 42, 43, 44, 45, 46, 47]. Furthermore, by performing differential gene analysis between the H‐IE as well as L‐IE groups and selecting top ranked genes (*n* = 100, 200, 300) for enrichment analysis, systemic immune function damage, extracellular matrix‐related factors, and disorders in lipid and protein metabolism likely contribute to the resistance in the H‐IE group (Figures S6F and S7I–J).

### 2.8 The Interaction Strength of the COL1A1‐COL1A2 and CCL5‐CXCR3 Receptor–Ligand Pairs Might Mediate the Efficacy of Immunotherapy at the Single‐Cell Level

**FIGURE 6:**
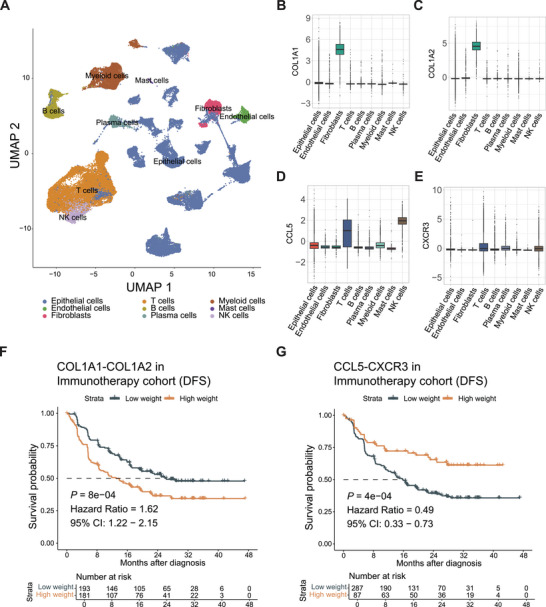
The interaction strength of the COL1A1‐COL1A2 and CCL5‐CXCR3 receptor–ligand pairs might mediate the efficacy of immunotherapy at the single‐cell level. (A) UMAP analysis of single cells from tissue samples of 12 urothelial cancer patients. In the UMAP map, nine cell‐type clusters are labeled by different colors. (B–E) The expression of receptor‒ligand pairs in various cell types were visualized in a bar plot at the single‐cell level. (F–G) Kaplan–Meier curves were generated to analyze DFS for receptor–ligand pairs in the immunotherapy set of IMvigor010 cohort.

Recent advancements in single‐cell technology have provided a comprehensive understanding of the fundamental properties of various cell types within TME. In this study, we also acquired single‐cell RNA sequencing data from 12 urothelial carcinoma patients who received ureterectomy or radical nephroureterectomy [48]. Subsequently, we standardized the data, resulting in the inclusion of 67,392 cells, which were assigned to nine major cell types based on gene expression markers, including T cells, myeloid cells, epithelial cells, fibroblasts, endothelial cells, mast cells, B cells, plasma cells, and natural killer (NK) cells (Figure 6A).

To further investigate cell interactions, we analyzed the expression of several crucial ligand–receptor pairs identified in the immunotherapy cohort across different cell types (Figure 6B–I). Members of the collagen family were commonly appeared in the low UAIscore group, such as collagen type I alpha 1 chain (COL1A1) and collagen type I alpha 2 chain (COL1A2) [49], which were significantly higher expressed in fibroblasts, suggesting a potential association between immunotherapy resistance and the active function of fibroblasts (Figure 6B,C). Conversely, receptor ligands with significantly stronger interactions in the high UAIscore group, such as C‐C motif chemokine ligand 5 (CCL5) and C‐X‐C motif chemokine receptor 3 (CXCR3), were prominently expressed in T cells and NK cells (Figure 6D,E). Previous studies have suggested that CCL5 produced by tumors and CXCR3 ligands induced by interferon‐gamma (IFN‐γ) from myeloid cells play a crucial role in facilitating T‐cell infiltration in immunoreactive tumors [50], indicating that enhanced interactions among these cells may contribute to a better response to immunotherapy in the high UAIscore group. Survival analysis of multiple receptor–ligand pairs in the immunotherapy cohort showed that the interaction strength of COL1A1‐COL1A2 was associated with poorer survival, whereas high CCL5‐CXCR3 interaction was associated with better outcomes, suggesting that these interactions might have mediated the benefit of immunotherapy (Figure 6F–G). In conclusion, the activity of receptor–ligand pair interactions, such as COL1A1‐COL1A2 in the low UAIscore group and CCL5‐CXCR3 in the high UAIscore group, might play different regulatory roles in the efficacy of immunotherapy.

## 3 Discussion

Numerous previous studies have utilized biomarkers derived from multiple‐omics to predict the efficacy of chemotherapy and immunotherapy based on genome, transcriptome, epigenome, and multi‐omics combinations in urothelial carcinoma [14, 15]. However, the application of these biomarkers in adjuvant immunotherapy has not yielded satisfactory results, and most studies have failed to establish a connection between data models and resistance mechanisms. ctDNA [7] has been proposed as an effective predictor of adjuvant immunotherapy in the follow‐up study of IMvigor010. It showed promising application prospects, but its clinical utilization is still limited due to high cost and measurement standardization. The UAIscore, a machine learning model developed based on transcriptomic data and guided by resistance mechanisms, is the first molecular biomarker for predicting adjuvant immunotherapy outcomes in urothelial carcinoma.

We evaluated the predictive ability of UAIscore and found that it performed exceptionally well in predicting survival and prognosis. Notably, UAIscore outperformed ctDNA, tTMB, and PD‐L1, which suggested that UAIscore could serve as an independent and excellent biomarker of immunotherapy. Furthermore, the predictive efficacy of the combination of ctDNA, tTMB, PD‐L1, and UAIscore was consistently superior to UAIscore alone in a period of time, highlighting the importance of integrating multi‐omic biomarkers. The results of multivariate analysis also presented that UAIscore had predictive value in both ctDNA‐positive and ctDNA‐negative groups. It enabled a more precise and personalized treatment plan and partially compensated for the predictive limitations of ctDNA when used in conjunction with the UAIscore for patient stratification. Specifically, patients with high UAIscore and positive ctDNA positive were more likely to respond to ICBs.

As for other clinical characteristics such as relapse status as well as tumor stage, respond and non‐respond patients performed significantly different. The factors contributing to immunotherapy efficacy have been the subject of numerous studies [39, 51, 52, 53], with immune infiltration often highlighted as a critical focus. As expected, a high degree of immune infiltration, including enrichment of cytotoxic T cells and increased PD‐1 expression in TME, was emphasized in most patients who responded to immunotherapy. Further analysis of TCGA subtype revealed that the luminal subtype displayed the highest level of immune infiltration and lowest degree of hypoxia may be a reason for the favorable prognosis and the benefits from immunotherapy. In addition to the immune infiltration, we also captured the involvement of specific resistant molecules, pathways, and receptor–ligand pairs that play crucial roles in determining treatment outcomes, which might remodel the tumor immune microenvironment. These findings have been partially validated at the single‐cell level. Therefore, combined with previous studies, the results of this study can provide a foundation and new ideas for a combination of immunotherapy strategies. For instance, targeting VEGFR can alleviate hypoxia and reduce immunosuppression, and anti‐angiogenesis drugs combined with immunotherapy have been proven in preclinical studies to produce more effective antitumor effects [32]. Insulin‐like growth factor 1 receptor (IGF‐1R), which can be inhibited by picropodophyllin (PPP), enhances the therapeutic efficacy of immunotherapy through blockading both immunogenic cytotoxins and PD‐1 [33]. Besides, FGF/FGFR promoted the survival of Treg cells and the M2 polarization of tumor‐associated macrophages (TAMs) by facilitating interleukin‐2 (IL‐2)‐mediated signal transducer and activator of transcription (STAT5) phosphorylation, thereby promoting an suppressive immune microenvironment [34]. Currently, a series of clinical studies combining immunotherapy with targeted therapy [54] and interventions that modify the local TME [55, 56] have been undertaken to enhance immunotherapy efficacy and address drug resistance.

Interestingly, our study revealed that a few patients did not show differences in prognosis based on variations in the degree of immune infiltration, whose contradictions create challenges for only TME stratification instructing immunotherapy. In previous literature, tumors that simultaneously exhibit PD‐L1 expression and TILs infiltration expected to respond to anti‐PD therapy but do not respond initially were defined as primary resistance [39]. Although the existence of TILs seems to be necessary, it is unclear which key cellular components dictate primary resistance [39]. Solving this issue, we revealed that the interaction between TNF‐α pathway and NF‐κB pathway led to immunotherapy resistance, potentially by inhibiting the function of immune cells within the immune‐enriched TME. Previous studies have shown that both TNF‐α and NF‐κB have a bidirectional effect on shaping the TME and on the occurrence and metastasis of tumors [41, 42, 43, 44, 45, 46, 47]. While TNF‐α can inhibit tumor cell growth by inducing apoptosis in cancer cells, it also stimulates the proliferation, survival, migration, and angiogenesis of most cancer cells that are resistant to TNF‐α induced cytotoxicity, leading to tumor development [41, 42, 43]. NF‐κB has long been considered a potential target for disease treatment. In addition to inhibiting key components of the NF‐κB pathway, targeting TNF‐α, which acts as an activator and effector of the NF‐κB pathway, is also expected to achieve the goal of suppressing tumor growth [44, 45, 46, 47]. In addition, the macroscopic tumor environment [57, 58] and intratumoral microbes [59] had to be considered, because their stability may improve immunotherapy outcomes in past studies.

In recent years, AI has demonstrated remarkable performance across various clinical fields and has also aided in capturing processes such as tumorigenesis, progression, and treatment resistance [13]. AI models can integrate multimodal data to reveal associations between different modalities, such as the relationship between pathology and the origin of primary tumors, the connections between multi‐omics features and cancer subtypes [13, 60, 61, 62], and the influence of metabolic signatures on immune dynamics. These associations can help identify new biomarkers or enhance the performance of existing ones, providing more accurate diagnostic and therapeutic predictions [63, 64]. However, prospective trial designs are still necessary to validate the effectiveness of these models, thus providing high‐quality evidence for widespread clinical use. In the future, our study could integrate multi‐omics data, including pathology, radiomics, and microbiomics, to improve the predictive performance of the UAIscore, explore the multi‐omics characteristics of patients stratified by different UAIscore from multiple perspectives, and uncover more specific resistance mechanisms to develop combined treatment strategies.

## 4 Limitations of the Study

Admittedly, this study does have certain limitations that must be acknowledged. The availability of publicly accessible datasets for adjuvant immunotherapy is currently limited, which hinders our ability to validate the predictive capability of UAIscore across different datasets thoroughly. Additionally, due to the limited types of omics data included in the IMvigor010 cohort, it was not possible to explore and identify more specific resistance mechanisms from a deeper perspective, thus providing higher level evidence for combined treatments that are most likely to enhance immunotherapy efficacy. We will continue to follow up on the immunotherapy cohort data to verify the predictive value of UAIscore and explore the mechanism of immunotherapy resistance in more independent cohorts.

## 5 Conclusion

Our study systematically analyzed the multi‐omics factors influencing adjuvant immunotherapy efficacy, leading to the development of the UAIscore model. Adjuvant atezolizumab may be associated with improved outcomes compared with observation in patients who are detected as having high UAIscore. The mechanisms of resistance were preliminarily explored, and a rationale for combination therapy was suggested in urothelial carcinoma.

## 6 Materials and Methods

### 6.1 Data Acquisition and Processing

This study was performed using tissue samples from the phase III, multicenter, open‐label, randomized trial IMvigor010 clinical (NCT02450331), which aimed to evaluate the efficacy of adjuvant immunotherapy in patients with histologically confirmed MIUC. Eligible patients had undergone radical surgical resection with lymph node dissection and exhibited no evidence of residual disease or metastasis, as confirmed by negative postoperative radiological imaging. Initially, 809 patients from 24 countries or regions were recruited, who were randomized in a 1:1 ratio to receive atezolizumab (1200 mg every 3 weeks) for up to 1 year as adjuvant therapy or undergo observation. A total of 728 patients met the clinical trial criteria, providing tissue samples for transcriptomic sequencing, which fully aligned with our research objectives and were entirely utilized for model construction and subsequent analyses. The primary efficacy endpoint was DFS, which was defined by local (pelvic) or urinary tract recurrence or distant urothelial carcinoma metastasis or death from any cause. The secondary efficacy endpoint was OS, which was defined as the time from randomization to death from any cause. The transcriptome data and primary clinical data of IMvigor010 trial are available after approval at the European genome‐phenome archive (EGA) (https://ega‐archive.org/datasets/, accession number: EGAS00001004997) [6]. The RNA‐sequencing data of the TCGA‐BLCA dataset were obtained from the TCGA data portal (https://portal.gdc.cancer.gov/) using the TCGAbiolinks R package in March 2022, and the clinical data were collected from the research of Liu J et al. [65]. The data used in this study are standardized Level 3 data (processed and aggregated results), which remain freely accessible on the TCGA website and are explicitly excluded from current US access restrictions. The independent single‐cell RNA‐seq datasets of 12 urothelial cancer patients who underwent ureterectomy or radical nephroureterectomy are publicly available through the Genome Sequence Archive in the National Genomics Data Center (NGDC) (https://ngdc.cncb.ac.cn/gsa‐human/browse/, accession number: HRA001867). [48]. All trials were conducted according to Good Clinical Practice and the Declaration of Helsinki. Protocol approval was obtained from independent review boards or ethics committees for each participating site, and informed consent was obtained from all patients. All data were accessed as per institutional protocols and jurisdictional compliance standards.

### 6.2 The UAIscore Conducted by the Random Forest Algorithm

The transcriptome data of 24,443 genes from 728 MIUC patients in the IMvigor010 clinical trial were analyzed. The median difference was used to remove 12,221 genes with low variation filtered by the median absolute deviation (MAD) method. A total of 728 patients were divided into a training group (*n* = 475) and a validation group (*n* = 253) according to 6.5:3.5 to further screen genes related to survival in all patients. Batch survival analysis was performed for DFS in the training group, and the 2259 genes associated with both DFS and overall survival were selected (*p* < 0.05). The mean of each gene expression level was taken as the cut‐off value, and the training group samples were divided into high and low UAIscore group. Subgroup survival analysis was performed in the immunotherapy and the observation groups using the Publish R package (https://github.com/tagteam/Publish), and the interaction genes whose expression level caused survival differences in only one treatment group were filtered out (*p* < 0.05). These survival interaction genes were divided into two parts with the boundary of HR = 1 and combined to obtain 91 marker genes for the final training of the random forest model. By means of data from immunotherapy patients in the training cohort, a risk score was generated based on the expression levels of selected marker genes, named the urothelial carcinoma adjuvant immunotherapy prediction score (UAIscore). The median UAIscore was used to stratify patients into high UAIscore and low UAIscore groups. The R package is available at https://github.com/LiaoWJLab/uaiscore.

### 6.3 Transcriptomic Data Processing

Whole‐transcriptome profiles were generated using TruSeq RNA Access technology (Illumina). Initially, quality control (QC) checks were performed on the raw sequence data using fastp (v.0.23.4), which provided insights into the quality metrics such as base quality, sequence quality, and the presence of adaptors. The trimming process ensured that only high‐quality, clean reads were used in subsequent analyses. For the alignment of the trimmed reads, we employed the STAR aligner (v.2.7.1a). Reads were mapped to the human reference genome GRCh38 (v111), utilizing the comprehensive gene annotations provided by the Gencode project (release 44). STAR was configured to perform a two‐pass alignment, which is particularly effective at detecting novel splice junctions and improving alignment rates. Post‐alignment, STAR also generated gene‐level quantification in the form of read counts per gene, using the –quantMode GeneCounts option. This output was crucial for downstream differential gene expression analysis. Finally, the read counts obtained from STAR were used to assemble a gene expression matrix.

### 6.4 Detection of ctDNA, tTMB, and PD‐L1

A total of 581 plasma samples were collected on the first day of the first treatment cycle (C1D1), while 495 peripheral blood samples were obtained on the first day of the third treatment cycle (C3D1). ctDNA detection was performed using Natera. After collection, plasma samples were processed within 30 min, separated, and stored at −80°C. Subsequent steps included cell‐free DNA (cfDNA) extraction and library preparation. For those samples where ctDNA was detected, the mean number of tumor molecules per milliliter of plasma was documented and calculated across all variants that met QC standards.

tTMB was evaluated based on the total number of somatic mutations per megabase of the captured exome. Patients whose tTMB was 10 or more mutations per megabase were considered tTMB‐positive, consistent with the average ctDNA BEP.

The central evaluation of PD‐L1 expression was conducted using the VENTANA SP142 immunohistochemistry assay. This assay employed a rabbit monoclonal primary antibody, which was diluted to a total protein concentration of 3 mg/mL. Tumors were classified as PD‐L1 expressing (IC2/3 status) when PD‐L1‐positive immune cells infiltrating the tumor accounted for 5% or more of the tumor area.

### 6.5 Multivariate Analysis

Patients were divided into the immunotherapy cohort and the observation cohort based on their treatment background in IMvigor010 clinical trial. A Cox proportional hazards model was utilized for multivariable regression analysis to assess the independent impact of the UAIscore on survival time, incorporating confounding factors such as gender, ctDNA status, tTMB status, PD‐L1 status, tumor stage, nodal status, number of lymph nodes resected, and phenotype.

### 6.6 Inference of Cell Fraction and Signature Score

We integrated TME cell type signature collected from several TME computational tools (CIBERSORT, MCP‐counter, ESTIMATE, EPIC, quantiseq, xCell) as well as the Immulo‐Oncology Biological Research (IOBR) R package (https://github.com/IOBR/IOBR) [38, 66] to estimate the infiltration of multiple cell types in the above dataset and subsequent TME clustering was performed based on the above results. Using the gene set variation analysis (GSVA) algorithm, we used the Gene Ontology (GO), Kyoto Encyclopedia of Genes and Genomes (KEGG), REACTOME, and HALLMARK gene sets to estimate the pathway enrichment score for each sample. The IOBR R package calculated scores for other popular genetic markers related to TME, tumor innate pathways, and metabolism for each sample. The Pathway RespOnsive GENes for activity Inference (PROGENy) R package (https://github.com/saezlab/progeny) was used to assess the activation level of 14 tumor signaling pathways.

### 6.7 Identification of TME Subtypes

Based on the TME cell type results obtained from signature calculations in xCell, MCP‐counter, EPIC, quantiseq, and IOBR (https://github.com/IOBR/IOBR) [38, 66], a consensus clustering algorithm was used to determine the number of clusters in the immunotherapy cohort in IMvigor010. This process is performed using the ConsensusClusterPlus R package (http://www.bioconductor.org/) and is repeated 1000 times to ensure classification stability. Unsupervised cluster analysis was performed on high and low UAIscore patients in the IO cohort, and the best discriminative power of each classification cluster was obtained when *k* was set at 2.

### 6.8 Statistical Analysis

The Shapiro–Wilk normality test was used to test the normality of the variables. For comparisons between two groups, the unpaired Student's *t*‐test was used to determine the statistical significance of normally distributed variables, and the Mann–Whitney *U* test (Wilcoxon rank sum test) was used to determine the statistical significance of nonnormally distributed variables. Kruskal–Wallis and one‐way ANOVA tests for nonparametric and parametric methods were used to compare two or more groups. Spearman and distance correlations were used to analyze correlations between normally distributed data. The chi‐squared test and bilateral Fisher exact test were used for contingency table analysis. Survival curves for each subgroup in the dataset were constructed using the Kaplan–Meier method, and the log‐rank (Mantel Cox) test was used to determine whether they were significantly different. A univariate Cox proportional hazards regression model was used to calculate the risk ratio from the univariate analysis. Multivariate Cox regression models were used to identify independent prognostic factors using the survminer R package. All statistical analyses were performed using R V.4.3.1 (https://www.r‐project.org/) with a two‐sided *p* value. A *p* value less than 0.05 was considered statistically significant. The *p* value adjusted for multiple testing was calculated using the Benjamini–Hochberg correction method.

## Author Contributions

**X.T.H**.: conceptualization, methodology, formal analysis, data curation, writing – original draft preparation, visualization. **W.J.Q**.: investigation, resources, data curation, project administration. **Y.Y.K**.: investigation, resources, data curation, project administration. **Q.Y.O**.: investigation, writing and original draft preparation. **Q.Q.M**.: formal analysis. **Y.R.F**: resources, investigation. **Z.Y.F**.: resources, investigation. **J.N.W**.: writing – original draft preparation. **X.S.L**.: writing – original draft preparation. **W.C.G**.: resources, supervision. **P.L**.: resources, project administration. **J.F.W**.: resources, project administration, funding acquisition. **J.P.B**.: resources, supervision. **Y.L.L**.: resources, visualization. **M.S**.: supervision, methodology. **Z.Q.W**.: supervision, project administration. **H.Y.S**.: project administration, methodology. **Y.F.Y**.: project administration, writing – review and editing, software, validation. **W.J.L**.: project administration, conceptualization, resources, supervision, writing – review and editing, funding acquisition. **D.Q.Z**.: project administration, conceptualization, methodology, writing – review and editing, software, validation. All authors have read and approved the final manuscript.

## Ethics Statement

The IMvigor010 study (ClinicalTrials.gov ID: NCT02450331), sponsored by Hoffmann‐La Roche, received ethical approval from institutional review boards/ethics committees at all participating sites. The HRA001867 dataset was approved by the Ethics Committee of Peking University First Hospital (Approval No. 2018[186]). All procedures involving human participants complied with the ethical standards of the institutional ethics committees and the Declaration of Helsinki. Informed consent was obtained from all participants or their legal representatives prior to sample collection.

## Conflicts of Interest

The authors declare no conflicts of interest.

## Supporting information




**Figure S1**: A flowchart delineating process of model building and Performance verification.
**Figure S2**: The predictive performance of multi‐omics biomarkers.
**Figure S3**: Multivariate Cox proportional hazard analyses of clinical variables and treatments for ctDNA subgroup.
**Figure S4**: Analysis of TCGA‐subtype in IMvigor010 clinical trial.
**Figure S5**: Benefit and resistance mechanism of immunotherapy cohort.
**Figure S6**: TNF‐á and the NF‐êB pathway may lead to resistance in immune‐enriched tumor microenvironments.
**Figure S7**: Clinical characteristics and enrichment of pathways in different tumor immune microenvironment.

**Supporting File**: mco270324‐sup‐0002‐Table.xlsx

## Data Availability

All datasets presented in this study can be found in online repositories. The data of IMvigor010 clinical trials (accession number: EGAS00001004997) was available after pass the application at the European Genome‐phenome Archive (EGA). The RNA‐sequencing data of the TCGA‐BLCA dataset were gained from the TCGA data portal (https://portal.gdc.cancer.gov/), and the clinical data were collected from the research of Liu J et al. [65]. The single‐cell RNA‐seq dataset was obtained from the National Genomics Data Center (CNCB; accession number: HRA001867). The data generated in this study are available in the article and its Supporting Information files. The R package is available at https://github.com/LiaoWJLab/uaiscore.
