# Supplementary material for: Artificial Intelligence‐Based Multimodal Prediction of Postoperative Adjuvant Immunotherapy Benefit in Urothelial Carcinoma: Results From the Phase III, Multicenter, Randomized, IMvigor010 Trial
Source: MedComm (2020). 2025 Aug 25;6(9):e70324. doi: 10.1002/mco2.70324 (PMC12377507; doi:10.1002/mco2.70324)
Supplement: Supplementary file 1 — Figure S1: A flowchart delineating process of model building and Performance verification. Figure S2: The predictive performance of multi‐omics biomarkers. Figure S3: Multivariate Cox proportional hazard analyses of clinical variables and treatments for ctDNA subgroup. Figure S4: Analysis of TCGA‐subtype in IMvigor010 clinical trial. Figure S5: Benefit and resistance mechanism of immunotherapy cohort. Figure S6: TNF‐á and the NF‐êB pathway may lead to resistance in immune‐enriched tumor microenvironments. Figure S7: Clinical characteristics and enrichment of pathways in different tumor immune microenvironment. [file MCO2-6-e70324-s001.pdf]

Supplementary Information: Materials and methods

1.1 | Assessing the predictive accuracy of different indicators

We used logistic regression analysis to calculate the result of combining two or more indicators. With 12, 24, and 36 months as time nodes, the ROC curve of a single indicator or a combined indicator was plotted. Its AUC value was calculated using the timeROC R package and compared for significant differences in AUC values between them. At the same time, the riskRegression R package (https://github.com/tagteam/riskRegression) was used to plot the continuous time-dependent AUC line plot and show its confidence interval.

1.2 | Functional and pathway enrichment analysis

The clusterProfiler R package (https://github.com/YuLab-SMU/clusterProfiler) was used for functional and pathway enrichment analysis of the samples. We analysed differentially expressed genes in the two groups, and adjusted $p < 0.05$ was used as the threshold of difference significance. The top 100, 200, and 300 genes were selected for GO and KEGG enrichment analysis to obtain the differentially expressed pathways and determine the stability of enrichment results. In addition, gene set enrichment analysis (GSEA) based on GO and KEGG gene sets was conducted on the adjusted expression data of all transcripts, with $|NES| > 1$, $p < 0.05$, and $FDR < 0.25$ as cut-off values. The up-down and down-down pathways in high-low UAIscore groups were identified and visualized.

1.3 | Cellular interaction estimation

The IMvigor010 immunotherapy cohort was evaluated using the Estimate Systems Immune Response (EaSIeR) R package (https://github.com/olapuentesantana/easier_manuscript), and the pairewise Wilcoxon test was used to identify differences in ligand-receptor interactions in the high UAIscore or low UAIscore groups. Survival analysis of multiple ligand-receptor pairs was performed, and a survival forest plot was generated.

1.4 | Identification of different subtypes of urothelial carcinoma

The BLCAsubtyping R package (https://github.com/cit-bioinfo/BLCAsubtyping) was used to determine the different molecular subtypes of patients in the IMvigor010 cohort. The categories include Baylor, UNC, MDA, CIT, Lund, and TCGA-subtypes.

1.5 | Single-cell RNA sequencing data processing

The raw unique molecular identifier (UMI) matrix was processed to filter out features detected in fewer than ten cells and cells with fewer than 300 genes. We further quantified the number of genes and UMIs for each cell. We retained high-quality cells with thresholds of 1000 UMIs, 300 genes, and less than 25% mitochondrial genes to ensure that most of the heterogeneous cell types were included in downstream analyses. The annotated information for the cells was taken from the supplementary information in the original article.

Supplementary Figures

Figure S1 A flowchart delineating process of model building and Performance verification

Figure S2 The predictive performance of multi-omics biomarkers

Figure S3 Multivariate Cox proportional hazard analyses of clinical variables and treatments for ctDNA subgroup

Figure S4 Analysis of TCGA-subtype in IMvigor010 clinical trial

Figure S5 Benefit and resistance mechanism of immunotherapy cohort

Figure S6 TNF-α and the NF-κB pathway may lead to resistance in immune-enriched tumor microenvironments

Figure S7 Clinical characteristics and enrichment of pathways in different tumor immune microenvironment

A

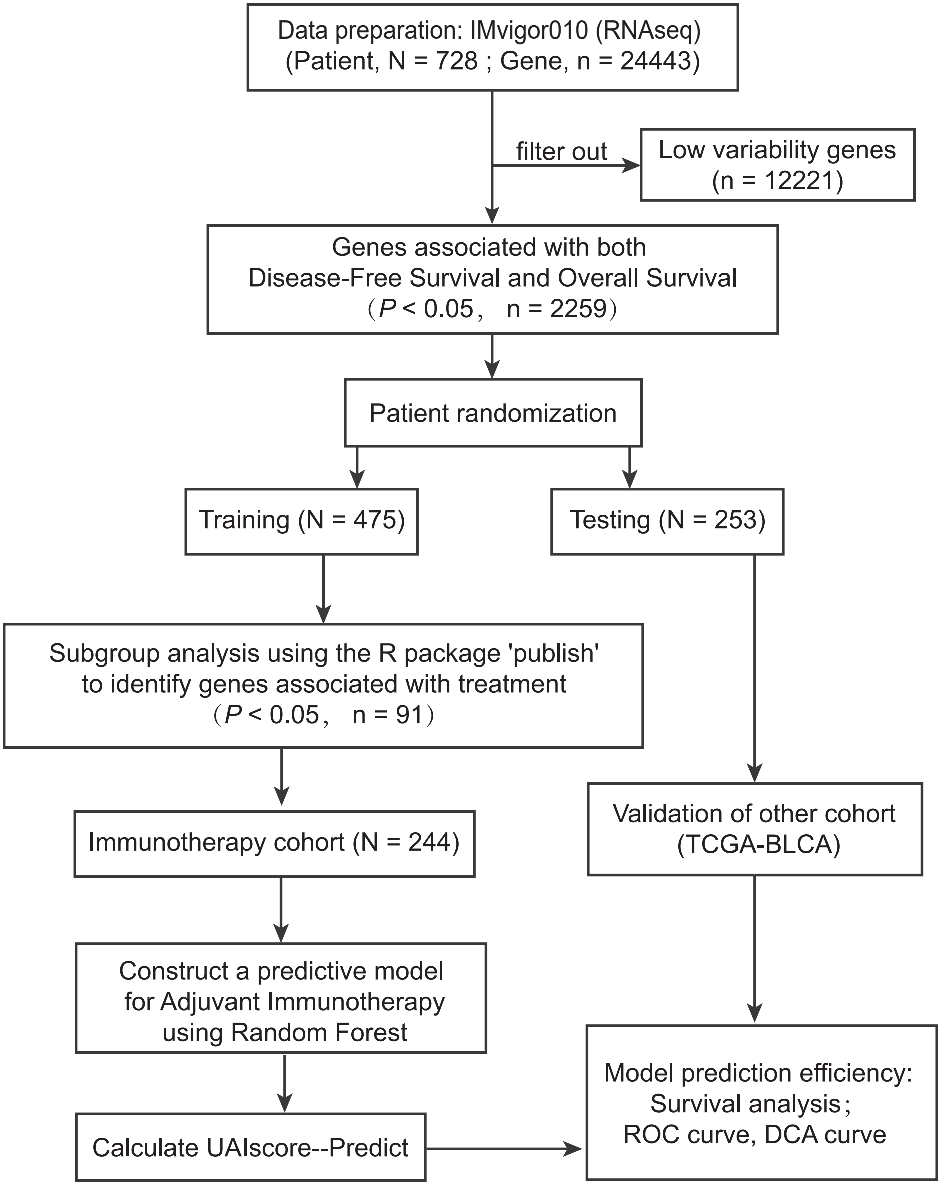

Data preparation: IMvigor010 (RNAseq)
(Patient, N = 728 ; Gene, n = 24443)
filter out
Low variability genes
(n = 12221)
Genes associated with both
Disease-Free Survival and Overall Survival
（P < 0.05， n = 2259）
Patient randomization
Training (N = 475)
Testing (N = 253)
Subgroup analysis using the R package 'publish'
to identify genes associated with treatment
（P < 0.05， n = 91）
Immunotherapy cohort (N = 244)
Construct a predictive model
for Adjuvant Immunotherapy
using Random Forest
Calculate UAIscore--Predict
Validation of other cohort
(TCGA-BLCA)
Model prediction efficiency:
Survival analysis；
ROC curve, DCA curve

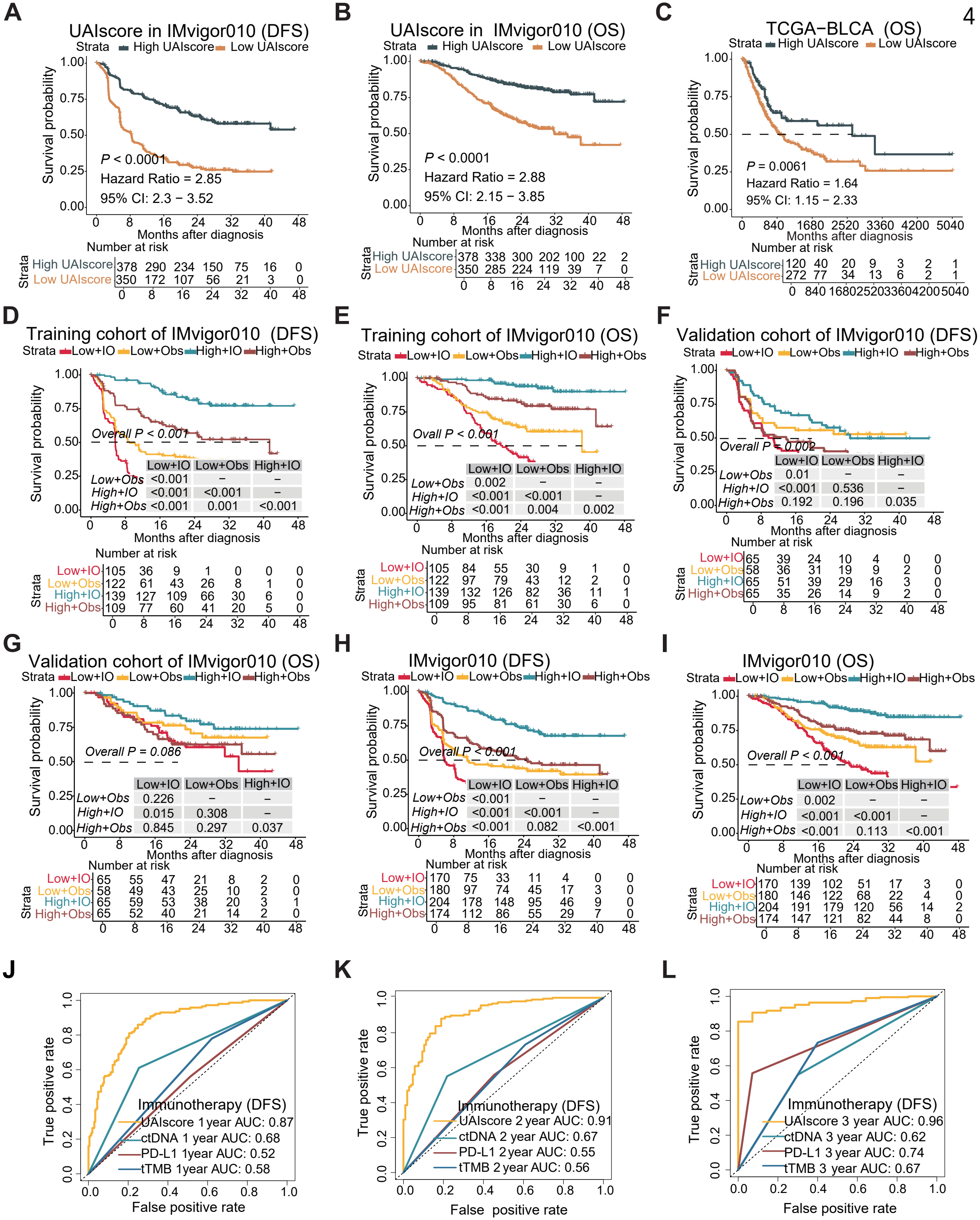
A
UAIscore in IMvigor010 (DFS)
Strata High UAIscore Low UAIscore
Survival probability
P < 0.0001
Hazard Ratio = 2.85
95% CI: 2.3 − 3.52
Months after diagnosis
Number at risk
High UAIscore 378 290 234 150 75 16 0
Low UAIscore 350 172 107 56 21 3 0
B
UAIscore in IMvigor010 (OS)
Strata High UAIscore Low UAIscore
P < 0.0001
Hazard Ratio = 2.88
95% CI: 2.15 − 3.85
Months after diagnosis
Number at risk
High UAIscore 378 338 300 202 100 22 2
Low UAIscore 350 285 224 119 39 7 0
C
TCGA−BLCA (OS)
Strata High UAIscore Low UAIscore
P = 0.0061
Hazard Ratio = 1.64
95% CI: 1.15 − 2.33
Months after diagnosis
Number at risk
High UAIscore 120 40 20 9 3 2 1
Low UAIscore 272 77 34 13 6 2 1
D
Training cohort of IMvigor010 (DFS)
Strata Low+IO Low+Obs High+IO High+Obs
Overall P < 0.001
Low+IO Low+Obs High+IO
Low+Obs <0.001 − −
High+IO <0.001 <0.001 −
High+Obs <0.001 0.001 <0.001
Number at risk
Low+IO 105 36 9 1 0 0 0
Low+Obs 122 61 43 26 8 1 0
High+IO 139 127 109 66 30 6 0
High+Obs 109 77 60 41 20 5 0
E
Training cohort of IMvigor010 (OS)
Strata Low+IO Low+Obs High+IO High+Obs
Ovall P < 0.001
Low+IO Low+Obs High+IO
Low+Obs 0.002 − −
High+IO <0.001 <0.001 −
High+Obs <0.001 0.004 0.002
Number at risk
Low+IO 105 84 55 30 9 1 0
Low+Obs 122 97 79 43 12 2 0
High+IO 139 132 126 82 36 11 1
High+Obs 109 95 81 61 30 6 0
F
Validation cohort of IMvigor010 (DFS)
Strata Low+IO Low+Obs High+IO High+Obs
Overall P = 0.002
Low+IO Low+Obs High+IO
Low+Obs 0.01 − −
High+IO <0.001 0.536 −
High+Obs 0.192 0.196 0.035
Number at risk
Low+IO 65 39 24 10 4 0 0
Low+Obs 58 36 31 19 9 2 0
High+IO 65 51 39 29 16 3 0
High+Obs 65 35 26 14 9 2 0
G
Validation cohort of IMvigor010 (OS)
Strata Low+IO Low+Obs High+IO High+Obs
Overall P = 0.086
Low+IO Low+Obs High+IO
Low+Obs 0.226 − −
High+IO 0.015 0.308 −
High+Obs 0.845 0.297 0.037
Number at risk
Low+IO 65 55 47 21 8 2 0
Low+Obs 58 49 43 25 10 2 0
High+IO 65 59 53 38 20 3 1
High+Obs 65 52 40 21 14 2 0
H
IMvigor010 (DFS)
Strata Low+IO Low+Obs High+IO High+Obs
Overall P < 0.001
Low+IO Low+Obs High+IO
Low+Obs <0.001 − −
High+IO <0.001 <0.001 −
High+Obs <0.001 0.082 <0.001
Number at risk
Low+IO 170 75 33 11 4 0 0
Low+Obs 180 97 74 45 17 3 0
High+IO 204 178 148 95 46 9 0
High+Obs 174 112 86 55 29 7 0
I
IMvigor010 (OS)
Strata Low+IO Low+Obs High+IO High+Obs
Overall P < 0.001
Low+IO Low+Obs High+IO
Low+Obs 0.002 − −
High+IO <0.001 <0.001 −
High+Obs <0.001 0.113 <0.001
Number at risk
Low+IO 170 139 102 51 17 3 0
Low+Obs 180 146 122 68 22 4 0
High+IO 204 191 179 120 56 14 2
High+Obs 174 147 121 82 44 8 0
J
True positive rate
False positive rate
Immunotherapy (DFS)
UAIscore 1year AUC: 0.87
ctDNA 1 year AUC: 0.68
PD-L1 1year AUC: 0.52
tTMB 1year AUC: 0.58
K
Immunotherapy (DFS)
UAIscore 2 year AUC: 0.91
ctDNA 2 year AUC: 0.67
PD-L1 2year AUC: 0.55
tTMB 2 year AUC: 0.56
L
Immunotherapy (DFS)
UAIscore 3 year AUC: 0.96
ctDNA 3 year AUC: 0.62
PD-L1 3 year AUC: 0.74
tTMB 3 year AUC: 0.67

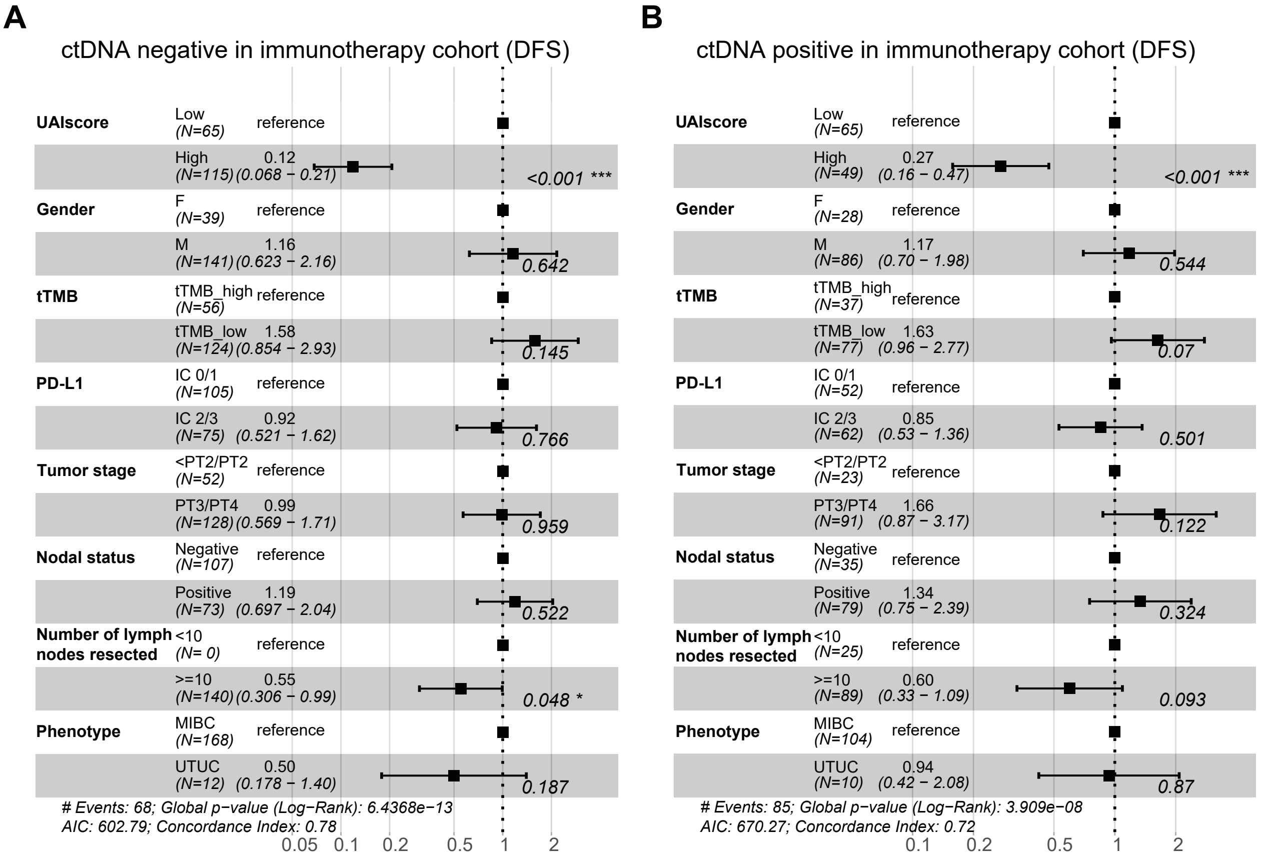
A
ctDNA negative in immunotherapy cohort (DFS)
UAIscore
Low (N=65) reference
High (N=115) 0.12 (0.068 − 0.21) <0.001 ***
Gender
F (N=39) reference
M (N=141) 1.16 (0.623 − 2.16) 0.642
tTMB
tTMB_high (N=56) reference
tTMB_low (N=124) 1.58 (0.854 − 2.93) 0.145
PD-L1
IC 0/1 (N=105) reference
IC 2/3 (N=75) 0.92 (0.521 − 1.62) 0.766
Tumor stage
<PT2/PT2 (N=52) reference
PT3/PT4 (N=128) 0.99 (0.569 − 1.71) 0.959
Nodal status
Negative (N=107) reference
Positive (N=73) 1.19 (0.697 − 2.04) 0.522
Number of lymph nodes resected
<10 (N= 0) reference
>=10 (N=140) 0.55 (0.306 − 0.99) 0.048 *
Phenotype
MIBC (N=168) reference
UTUC (N=12) 0.50 (0.178 − 1.40) 0.187
Events: 68; Global p−value (Log−Rank): 6.4368e−13
AIC: 602.79; Concordance Index: 0.78
0.05 0.1 0.2 0.5 1 2
B
ctDNA positive in immunotherapy cohort (DFS)
UAIscore
Low (N=65) reference
High (N=49) 0.27 (0.16 − 0.47) <0.001 ***
Gender
F (N=28) reference
M (N=86) 1.17 (0.70 − 1.98) 0.544
tTMB
tTMB_high (N=37) reference
tTMB_low (N=77) 1.63 (0.96 − 2.77) 0.07
PD-L1
IC 0/1 (N=52) reference
IC 2/3 (N=62) 0.85 (0.53 − 1.36) 0.501
Tumor stage
<PT2/PT2 (N=23) reference
PT3/PT4 (N=91) 1.66 (0.87 − 3.17) 0.122
Nodal status
Negative (N=35) reference
Positive (N=79) 1.34 (0.75 − 2.39) 0.324
Number of lymph nodes resected
<10 (N=25) reference
>=10 (N=89) 0.60 (0.33 − 1.09) 0.093
Phenotype
MIBC (N=104) reference
UTUC (N=10) 0.94 (0.42 − 2.08) 0.87
Events: 85; Global p−value (Log−Rank): 3.909e−08
AIC: 670.27; Concordance Index: 0.72
0.1 0.2 0.5 1 2

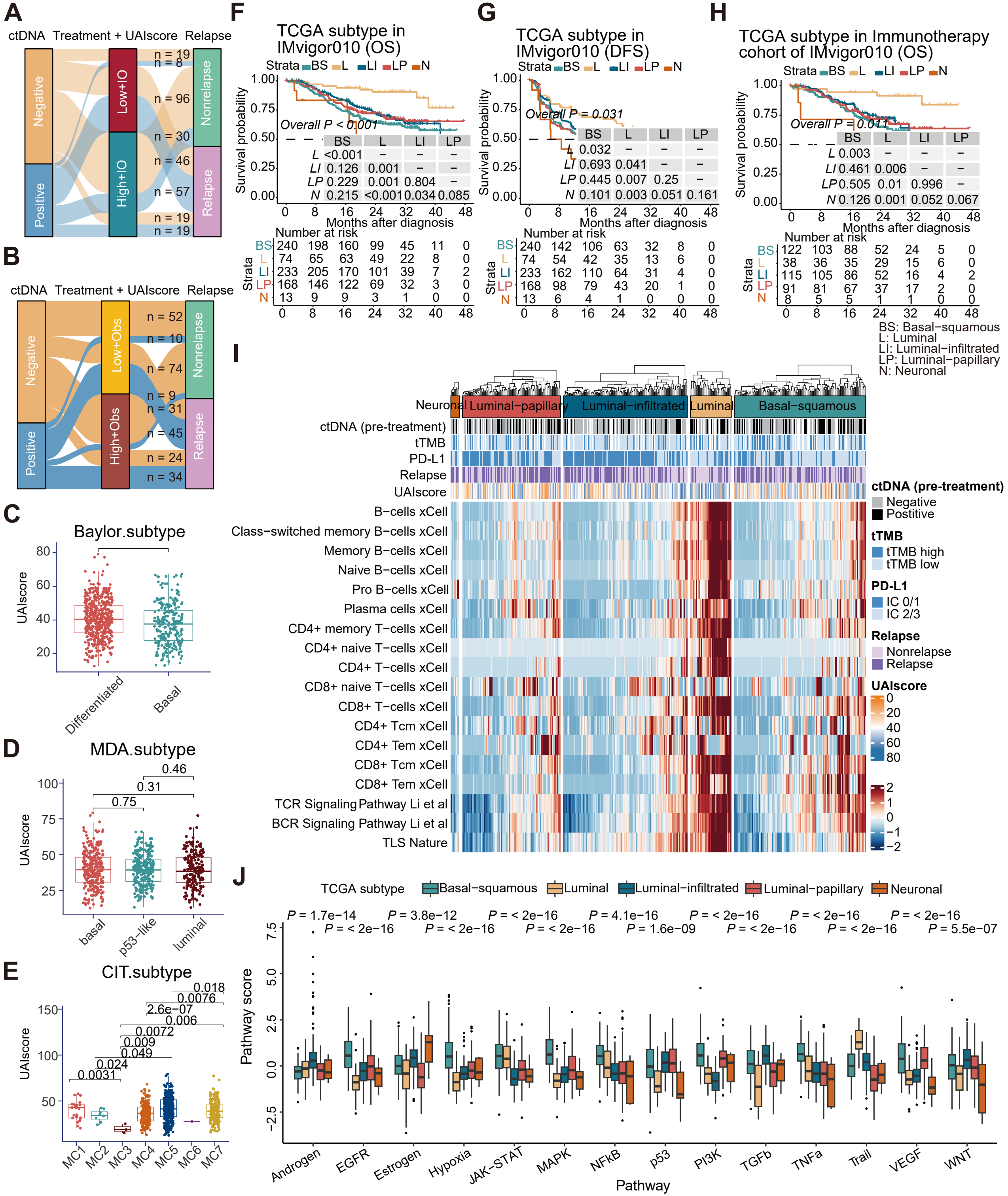
A
ctDNA Treatment + UAIscore Relapse
Negative
Positive
Low+IO
High+IO
Nonrelapse
Relapse
n = 19
n = 8
n = 96
n = 30
n = 46
n = 57
n = 19
n = 19
B
ctDNA Treatment + UAIscore Relapse
Negative
Positive
Low+Obs
High+Obs
Nonrelapse
Relapse
n = 52
n = 10
n = 74
n = 9
n = 31
n = 45
n = 24
n = 34
C
Baylor.subtype
UAIscore
Differentiated
Basal
D
MDA.subtype
UAIscore
0.46
0.31
0.75
basal
p53−like
luminal
E
CIT.subtype
UAIscore
0.018
0.0076
2.6e−07
0.006
0.0072
0.009
0.049
0.024
0.0031
MC1 MC2 MC3 MC4 MC5 MC6 MC7
F
TCGA subtype in IMvigor010 (OS)
Strata BS L LI LP N
Survival probability
Overall P < 0.001
BS L LI LP
L <0.001 − − −
LI 0.126 0.001 − −
LP 0.229 0.001 0.804 −
N 0.215 <0.001 0.034 0.085
Months after diagnosis
Number at risk
BS 240 198 160 99 45 11 0
L 74 65 63 49 22 8 0
LI 233 205 170 101 39 7 2
LP 168 146 122 69 32 3 0
N 13 9 9 3 1 0 0
Strata
G
TCGA subtype in IMvigor010 (DFS)
Strata BS L LI LP N
Survival probability
Overall P = 0.031
BS L LI LP
L 0.032 − − −
LI 0.693 0.041 − −
LP 0.445 0.007 0.25 −
N 0.101 0.003 0.051 0.161
Months after diagnosis
Number at risk
BS 240 142 106 63 32 8 0
L 74 54 42 35 13 6 0
LI 233 162 110 64 31 4 0
LP 168 98 79 43 20 1 0
N 13 6 4 1 0 0 0
Strata
H
TCGA subtype in Immunotherapy cohort of IMvigor010 (OS)
Strata BS L LI LP N
Survival probability
Overall P = 0.011
BS L LI LP
L 0.003 − − −
LI 0.461 0.006 − −
LP 0.505 0.01 0.996 −
N 0.126 0.001 0.052 0.067
Months after diagnosis
Number at risk
BS 122 103 88 52 24 5 0
L 38 36 35 29 15 6 0
LI 115 105 86 52 16 4 2
LP 91 81 67 37 17 2 0
N 8 5 5 1 1 0 0
Strata
BS: Basal−squamous
L: Luminal
LI: Luminal−infiltrated
LP: Luminal−papillary
N: Neuronal
I
Neuronal Luminal−papillary Luminal−infiltrated Luminal Basal−squamous
ctDNA (pre-treatment)
tTMB
PD-L1
Relapse
UAIscore
B−cells xCell
Class−switched memory B−cells xCell
Memory B−cells xCell
Naive B−cells xCell
Pro B−cells xCell
Plasma cells xCell
CD4+ memory T−cells xCell
CD4+ naive T−cells xCell
CD4+ T−cells xCell
CD8+ naive T−cells xCell
CD8+ T−cells xCell
CD4+ Tcm xCell
CD4+ Tem xCell
CD8+ Tcm xCell
CD8+ Tem xCell
TCR Signaling Pathway Li et al
BCR Signaling Pathway Li et al
TLS Nature
ctDNA (pre-treatment)
Negative
Postitive
tTMB
tTMB high
tTMB low
PD-L1
IC 0/1
IC 2/3
Relapse
Nonrelapse
Relapse
UAIscore
J
TCGA subtype Basal−squamous Luminal Luminal−infiltrated Luminal−papillary Neuronal
P = 1.7e−14
P = < 2e−16
P = 3.8e−12
P = < 2e−16
P = < 2e−16
P = < 2e−16
P = 4.1e−16
P = 1.6e−09
P = < 2e−16
P = < 2e−16
P = < 2e−16
P = < 2e−16
P = < 2e−16
P = 5.5e−07
Pathway score
Androgen EGFR Estrogen Hypoxia JAK−STAT MAPK NFkB p53 PI3K TGFb TNFa Trail VEGF WNT
Pathway

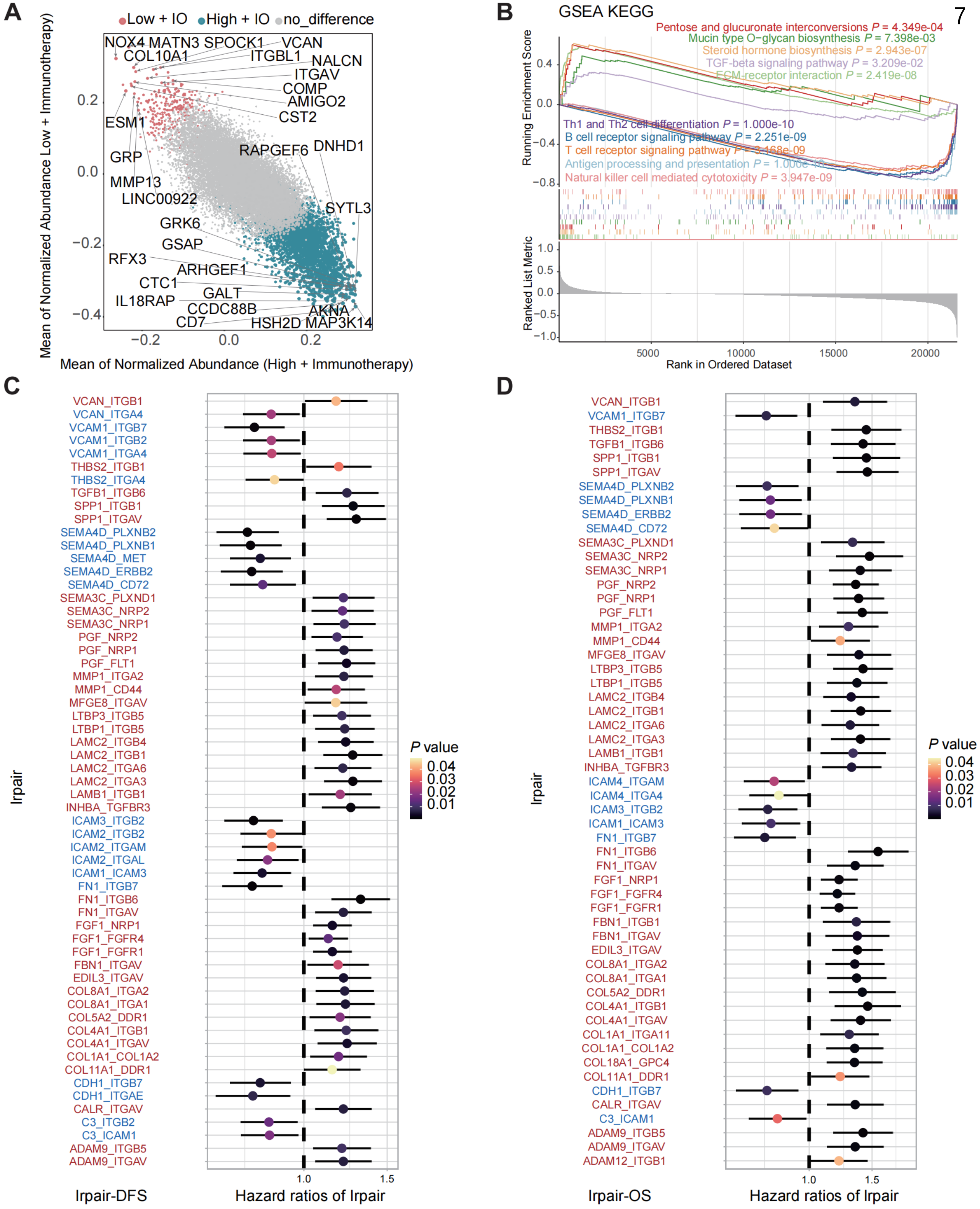
A
Low + IO
High + IO
no_difference
NOX4
MATN3
SPOCK1
VCAN
COL10A1
ITGBL1
NALCN
ITGAV
COMP
AMIGO2
CST2
ESM1
GRP
MMP13
LINC00922
RAPGEF6
DNHD1
SYTL3
GRK6
GSAP
RFX3
ARHGEF1
CTC1
GALT
IL18RAP
CCDC88B
AKNA
CD7
HSH2D
MAP3K14
Mean of Normalized Abundance Low + Immunotherapy)
Mean of Normalized Abundance (High + Immunotherapy)
0.2
0.0
−0.2
−0.4
B
GSEA KEGG
Pentose and glucuronate interconversions P = 4.349e-04
Mucin type O−glycan biosynthesis P = 7.398e-03
Steroid hormone biosynthesis P = 2.943e-07
TGF-beta signaling pathway P = 3.209e-02
ECM-receptor interaction P = 2.419e-08
Th1 and Th2 cell differentiation P = 1.000e-10
B cell receptor signaling pathway P = 2.251e-09
T cell receptor signaling pathway P = 3.168e-09
Antigen processing and presentation P = 1.000e-10
Natural killer cell mediated cytotoxicity P = 3.947e-09
Running Enrichment Score
Ranked List Metric
Rank in Ordered Dataset
5000
10000
15000
20000
C
VCAN_ITGB1
VCAN_ITGA4
VCAM1_ITGB7
VCAM1_ITGB2
VCAM1_ITGA4
THBS2_ITGB1
THBS2_ITGA4
TGFB1_ITGB6
SPP1_ITGB1
SPP1_ITGAV
SEMA4D_PLXNB2
SEMA4D_PLXNB1
SEMA4D_MET
SEMA4D_ERBB2
SEMA4D_CD72
SEMA3C_PLXND1
SEMA3C_NRP2
SEMA3C_NRP1
PGF_NRP2
PGF_NRP1
PGF_FLT1
MMP1_ITGA2
MMP1_CD44
MFGE8_ITGAV
LTBP3_ITGB5
LTBP1_ITGB5
LAMC2_ITGB4
LAMC2_ITGB1
LAMC2_ITGA6
LAMC2_ITGA3
LAMB1_ITGB1
INHBA_TGFBR3
ICAM3_ITGB2
ICAM2_ITGB2
ICAM2_ITGAM
ICAM2_ITGAL
ICAM1_ICAM3
FN1_ITGB7
FN1_ITGB6
FN1_ITGAV
FGF1_NRP1
FGF1_FGFR4
FGF1_FGFR1
FBN1_ITGAV
EDIL3_ITGAV
COL8A1_ITGA2
COL8A1_ITGA1
COL5A2_DDR1
COL4A1_ITGB1
COL4A1_ITGAV
COL1A1_COL1A2
COL11A1_DDR1
CDH1_ITGB7
CDH1_ITGAE
CALR_ITGAV
C3_ITGB2
C3_ICAM1
ADAM9_ITGB5
ADAM9_ITGAV
lrpair
P value
0.04
0.03
0.02
0.01
1.0
1.5
lrpair-DFS
Hazard ratios of lrpair
D
VCAN_ITGB1
VCAM1_ITGB7
THBS2_ITGB1
TGFB1_ITGB6
SPP1_ITGB1
SPP1_ITGAV
SEMA4D_PLXNB2
SEMA4D_PLXNB1
SEMA4D_ERBB2
SEMA4D_CD72
SEMA3C_PLXND1
SEMA3C_NRP2
SEMA3C_NRP1
PGF_NRP2
PGF_NRP1
PGF_FLT1
MMP1_ITGA2
MMP1_CD44
MFGE8_ITGAV
LTBP3_ITGB5
LTBP1_ITGB5
LAMC2_ITGB4
LAMC2_ITGB1
LAMC2_ITGA6
LAMC2_ITGA3
LAMB1_ITGB1
INHBA_TGFBR3
ICAM4_ITGAM
ICAM4_ITGA4
ICAM3_ITGB2
ICAM1_ICAM3
FN1_ITGB7
FN1_ITGB6
FN1_ITGAV
FGF1_NRP1
FGF1_FGFR4
FGF1_FGFR1
FBN1_ITGB1
FBN1_ITGAV
EDIL3_ITGAV
COL8A1_ITGA2
COL8A1_ITGA1
COL5A2_DDR1
COL4A1_ITGB1
COL4A1_ITGAV
COL1A1_ITGA11
COL1A1_COL1A2
COL18A1_GPC4
COL11A1_DDR1
CDH1_ITGB7
CALR_ITGAV
C3_ICAM1
ADAM9_ITGB5
ADAM9_ITGAV
ADAM12_ITGB1
lrpair
P value
0.04
0.03
0.02
0.01
1.0
1.5
lrpair-OS
Hazard ratios of lrpair

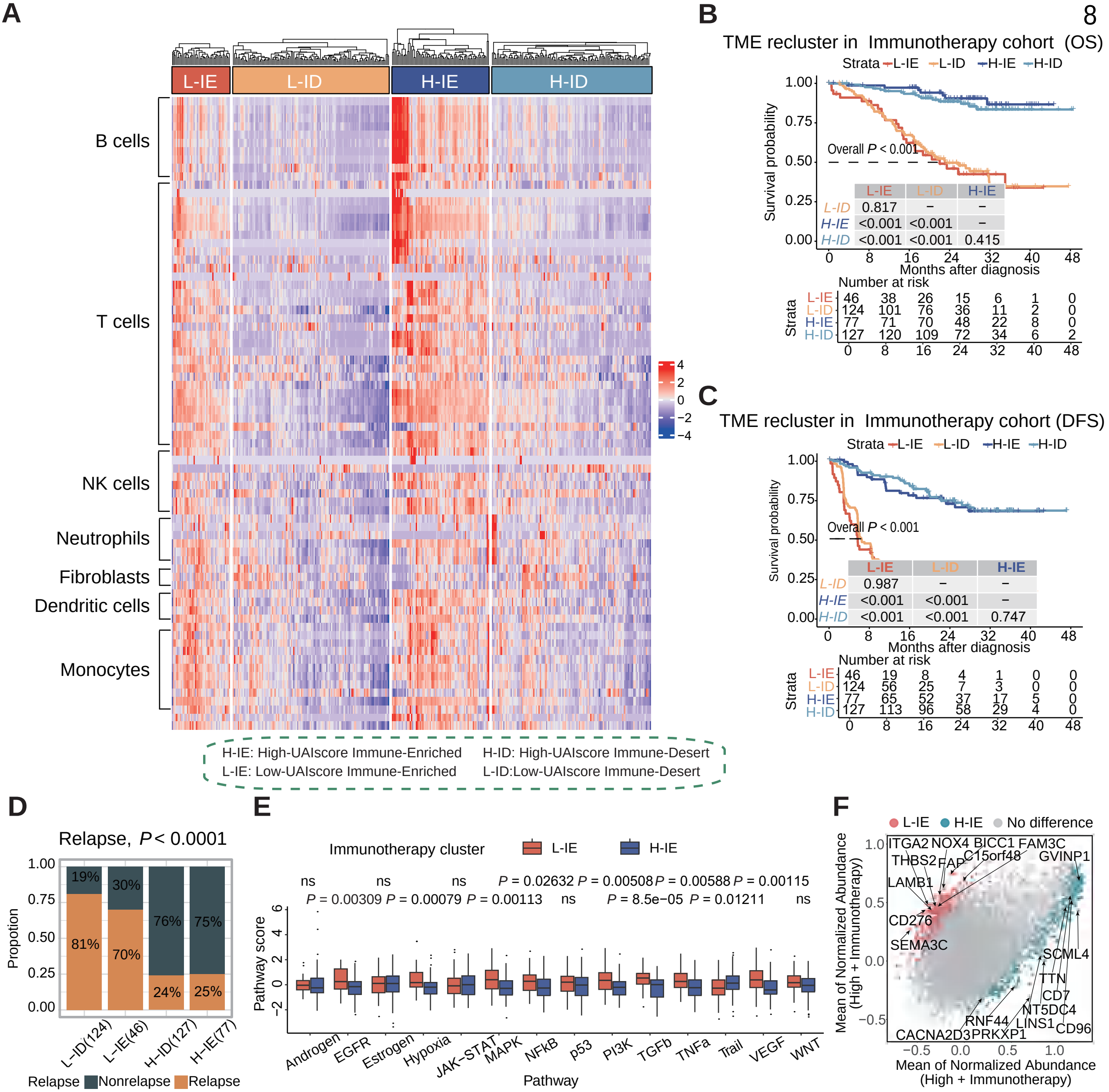
A
L-IE
L-ID
H-IE
H-ID
B cells
T cells
NK cells
Neutrophils
Fibroblasts
Dendritic cells
Monocytes
4
2
0
−2
−4
H-IE: High-UAIscore Immune-Enriched
L-IE: Low-UAIscore Immune-Enriched
H-ID: High-UAIscore Immune-Desert
L-ID:Low-UAIscore Immune-Desert
B
TME recluster in Immunotherapy cohort (OS)
Strata L-IE L-ID H-IE H-ID
Survival probability
1.00
0.75
0.50
0.25
0.00
Overall P < 0.001
L-IE L-ID H-IE
L-ID 0.817 – –
H-IE <0.001 <0.001 –
H-ID <0.001 <0.001 0.415
0 8 16 24 32 40 48
Months after diagnosis
Number at risk
Strata
L-IE 46 38 26 15 6 1 0
L-ID 124 101 76 36 11 2 0
H-IE 77 71 70 48 22 8 0
H-ID 127 120 109 72 34 6 2
0 8 16 24 32 40 48
C
TME recluster in Immunotherapy cohort (DFS)
Strata L-IE L-ID H-IE H-ID
Survival probability
1.00
0.75
0.50
0.25
0.00
Overall P < 0.001
L-IE L-ID H-IE
L-ID 0.987 – –
H-IE <0.001 <0.001 –
H-ID <0.001 <0.001 0.747
0 8 16 24 32 40 48
Months after diagnosis
Number at risk
Strata
L-IE 46 19 8 4 1 0 0
L-ID 124 56 25 7 3 0 0
H-IE 77 65 52 37 17 5 0
H-ID 127 113 96 58 29 4 0
0 8 16 24 32 40 48
D
Relapse, P < 0.0001
Propotion
1.00
0.75
0.50
0.25
0.00
19%
81%
30%
70%
76%
24%
75%
25%
L-ID(124)
L-IE(46)
H-ID(127)
H-IE(77)
Relapse Nonrelapse Relapse
E
Immunotherapy cluster L-IE H-IE
ns ns ns P = 0.02632 P = 0.00508 P = 0.00588 P = 0.00115
P = 0.00309 P = 0.00079 P = 0.00113 ns P = 8.5e−05 P = 0.01211 ns
Pathway score
6
4
2
0
−2
Androgen
EGFR
Estrogen
Hypoxia
JAK−STAT
MAPK
NFkB
p53
PI3K
TGFb
TNFa
Trail
VEGF
WNT
Pathway
F
L-IE H-IE No difference
Mean of Normalized Abundance (High + Immunotherapy)
1.0
0.5
0.0
−0.5
ITGA2
NOX4
BICC1
FAM3C
THBS2
FAP
C15orf48
GVINP1
LAMB1
CD276
SEMA3C
SCML4
TTN
CD7
NT5DC4
RNF44
LINS1
CD96
CACNA2D3
PRKXP1
−0.5 0.0 0.5 1.0
Mean of Normalized Abundance
(High + Immunotherapy)

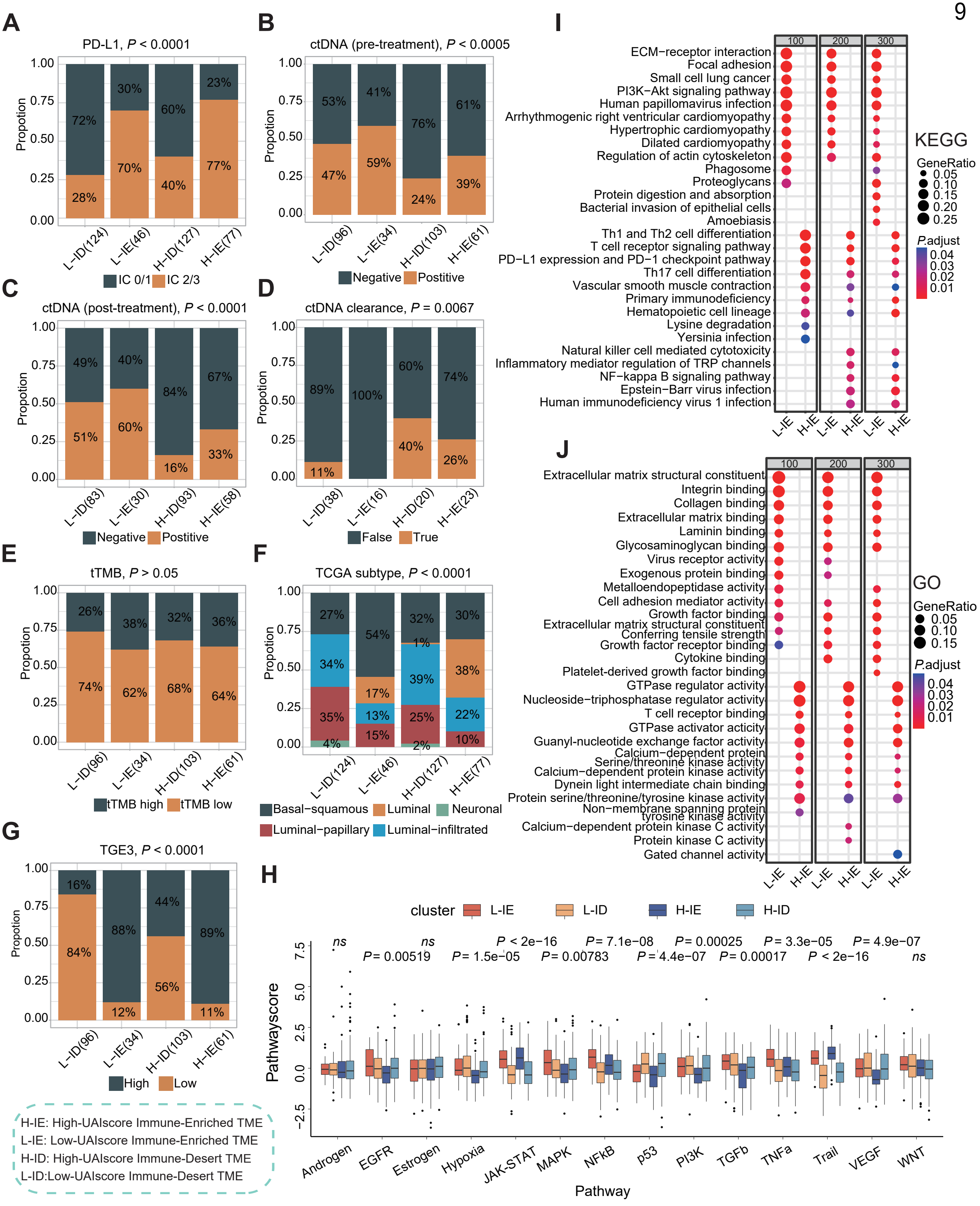
A
PD-L1, P < 0.0001
Propotion
L−ID(124) L−IE(46) H−ID(127) H−IE(77)
72% 28% 30% 70% 60% 40% 23% 77%
IC 0/1 IC 2/3
B
ctDNA (pre-treatment), P < 0.0005
L−ID(96) L−IE(34) H−ID(103) H−IE(61)
53% 47% 41% 59% 76% 24% 61% 39%
Negative Postitive
C
ctDNA (post-treatment), P < 0.0001
L−ID(83) L−IE(30) H−ID(93) H−IE(58)
49% 51% 40% 60% 84% 16% 67% 33%
Negative Postitive
D
ctDNA clearance, P = 0.0067
L−ID(38) L−IE(16) H−ID(20) H−IE(23)
89% 11% 100% 60% 40% 74% 26%
False True
E
tTMB, P > 0.05
L−ID(96) L−IE(34) H−ID(103) H−IE(61)
26% 74% 38% 62% 32% 68% 36% 64%
tTMB high tTMB low
F
TCGA subtype, P < 0.0001
L−ID(124) L−IE(46) H−ID(127) H−IE(77)
27% 34% 35% 4% 54% 17% 13% 15% 32% 1% 39% 25% 2% 30% 38% 22% 10%
Basal−squamous Luminal Neuronal
Luminal−papillary Luminal−infiltrated
G
TGE3, P < 0.0001
L−ID(96) L−IE(34) H−ID(103) H−IE(61)
16% 84% 88% 12% 44% 56% 89% 11%
High Low
H-IE: High-UAIscore Immune-Enriched TME
L-IE: Low-UAIscore Immune-Enriched TME
H-ID: High-UAIscore Immune-Desert TME
L-ID:Low-UAIscore Immune-Desert TME
I
100 200 300
ECM−receptor interaction
Focal adhesion
Small cell lung cancer
PI3K−Akt signaling pathway
Human papillomavirus infection
Arrhythmogenic right ventricular cardiomyopathy
Hypertrophic cardiomyopathy
Dilated cardiomyopathy
Regulation of actin cytoskeleton
Phagosome
Proteoglycans
Protein digestion and absorption
Bacterial invasion of epithelial cells
Amoebiasis
Th1 and Th2 cell differentiation
T cell receptor signaling pathway
PD−L1 expression and PD−1 checkpoint pathway
Th17 cell differentiation
Vascular smooth muscle contraction
Primary immunodeficiency
Hematopoietic cell lineage
Lysine degradation
Yersinia infection
Natural killer cell mediated cytotoxicity
Inflammatory mediator regulation of TRP channels
NF−kappa B signaling pathway
Epstein−Barr virus infection
Human immunodeficiency virus 1 infection
L−IE H−IE L−IE H−IE L−IE H−IE
KEGG
GeneRatio 0.05 0.10 0.15 0.20 0.25
P.adjust 0.04 0.03 0.02 0.01
J
100 200 300
Extracellular matrix structural constituent
Integrin binding
Collagen binding
Extracellular matrix binding
Laminin binding
Glycosaminoglycan binding
Virus receptor activity
Exogenous protein binding
Metalloendopeptidase activity
Cell adhesion mediator activity
Growth factor binding
Extracellular matrix structural constituent
Conferring tensile strength
Growth factor receptor binding
Cytokine binding
Platelet-derived growth factor binding
GTPase regulator activity
Nucleoside−triphosphatase regulator activity
T cell receptor binding
GTPase activator acticity
Guanyl-nucleotide exchange factor activity
Calcium−dependent protein
Serine/threonine kinase activity
Calcium−dependent protein kinase activity
Dynein light intermediate chain binding
Protein serine/threonine/tyrosine kinase activity
Non−membrane spanning protein
tyrosine kinase activity
Calcium−dependent protein kinase C activity
Protein kinase C activity
Gated channel activity
L−IE H−IE L−IE H−IE L−IE H−IE
GO
GeneRatio 0.05 0.10 0.15
P.adjust 0.04 0.03 0.02 0.01
H
cluster L−IE L−ID H−IE H−ID
ns P= 0.00519 ns P= 1.5e−05 P < 2e−16 P= 0.00783 P= 7.1e−08 P= 4.4e−07 P= 0.00025 P= 0.00017 P= 3.3e−05 P < 2e−16 P= 4.9e−07 ns
Pathwayscore
Androgen EGFR Estrogen Hypoxia JAK-STAT MAPK NFkB p53 PI3K TGFb TNFa Trail VEGF WNT
Pathway

The figure legend of Supplementary figures

Figure S1 A flowchart delineating process of model building and Performance verification

Figure S2 The predictive performance of multi-omics biomarkers

(A-B) Kaplan-Meier curves were generated to analyse DFS and OS for all patients in the IMvigor010 cohort. (C) Kaplan-Meier curves were generated to analyse OS in the TCGA-BLCA dataset. (D-G) Kaplan-Meier curves were generated to analyse DFS and OS in the training and validation sets of IMvigor010 cohort. The analysis was based on four subgroups categorized by the combination of UAIscore and treatment modality. (H-I) Kaplan-Meier curves of the combination of treatment and UAIscore were generated based on DFS and OS survival analysis in IMvigor010. (J-L) The ROC curve was used to assess the predictive efficacy of UAIscore, ctDNA (pre-treatment), tTMB, PD-L1 and combination on DFS in the immunotherapy cohort. tTMB, Tumor mutation burden; PD-L1, Programmed cell death-ligand 1; high, high UAIscore; low, low UAIscore; IO, immunotherapy; Obs, observation.

Figure S3 Multivariate Cox proportional hazard analyses of clinical variables and treatments for ctDNA subgroup

(A) Multivariate analysis of the ctDNA negative group in the immunotherapy cohorts. (B) Multivariate analysis of the ctDNA positive group in the immunotherapy cohorts. *, $p < 0.05$; **, $p < 0.01$; ***, $p < 0.001$. tTMB, Tumor mutation burden; PD-L1, Programmed cell death-ligand 1.

Figure S4 Analysis of TCGA-subtype in IMvigor010 clinical trial

(A-B) Sankey plots demonstrate the relationship between ctDNA (pretherapy), UAIscore, and recurrence in the immunotherapy (I) and observation (J) cohorts, respectively. (C-E) The BLCAsubtyping R package was employed to determine different molecular subtypes within the IMvigor010 cohort. The distribution of UAIscore among different subtype samples within each type was illustrated using box plots. The Wilcoxon test was conducted to analyse the differences in UAIscore between the two subtypes. (F-H) Kaplan-Meier curves were generated to perform survival analysis based on DFS and OS for the TCGA-subtype in both the immunotherapy and observation cohorts. (I) The tumor immune microenvironment of patients in the immunotherapy cohort was analysed using the tumor microenvironment signature from the IOBR package and xCell tumor immune invasion analysis tool. The distribution of immune microenvironment characteristics within the TCGA-subtype was presented through heatmap. (J) The PROGENy R package was employed to assess the degree of activation of 14 tumor signaling pathways in immunotherapy patients. The activation differences within the TCGA-subtype were displayed using box plots. *, $p < 0.05$; **, $p < 0.01$; ***, $p < 0.001$; ****, $p < 0.0001$; NS: $p > 0.05$. high, high UAIscore; low, low UAIscore; IO, immunotherapy; Obs, observation; tTMB, Tumor mutation burden; PD-L1, Programmed cell death-ligand 1.

Figure S5 Supplementary validation of core genes, pathways and ligand-receptor networks

(A) Gene differential expression analysis was conducted in immunotherapy patients using the Wilcoxon test to select the top 15 genes with significant expression differences between the high and low UAIscore groups based on adjusted p values. (B) In the immunotherapy cohort, genes

were ranked by the relative expression between the two groups, GSEA enrichment analysis was performed based on KEGG gene sets, and some representative pathways were selected among the pathways with a p value<0.05. (C-D) Representative ligand receptor pairs with significant differences between the high and low UAIscore groups were selected in immunotherapy cohort. Risk assessment of these ligand-receptor pairs was performed, highlighting a subset with p value < 0.05. GSEA, Gene set enrichment analysis; GO, Gene Ontology; KEGG, Kyoto Encyclopedia of Genes and Genomes.

Figure S6 The TNF-α/NF-κB signaling was associated with immunotherapy resistance in Immune- Enriched TME

(A) Heatmap showed the distribution of tumor immune microenvironment (TIME) signatures for further clustering in the four groups of the immunotherapy cohort. (B-C) In the immunotherapy cohort, patients were stratified into four groups based on their immune infiltration levels. Specifically, the H-IE and L-IE groups exhibited high levels of immune infiltration, while the H-ID and L-ID groups showed poor immune infiltration. Survival analysis was conducted on these four groups. (D) The bar plot provides the specific percentage of relapse status that show significant differences among the four groups. The statistical analysis was conducted using chi-square test. (E) Demonstration of the degree of activation of 14 tumor signaling pathways in H-IE and L-IE subgroups of the immunotherapy cohort in the bar plot. (F) Gene differential expression analysis with the Wilcoxon test was performed in the H-Ir and L-Ir groups of immunotherapy cohort, and the top 10 genes with significant expression differences in the two groups were selected based on adjusted p value. *, $p < 0.05$; **, $p < 0.01$; ***, $p < 0.001$; ****, $p < 0.0001$; NS: $p > 0.05$. H-IE, High-UAIscore Immune-Enriched TME; L-IE, Low-UAIscore Immune-Enriched TME; H-ID, High-UAIscore Immune-Desert TME; L-ID, Low-UAIscore Immune-Desert TME.

Figure S7 Clinical characteristics and enrichment of pathways in different tumor immune microenvironment

(A-G) The bar plot provides the specific percentage of important markers that show significant differences among the four groups. These markers include PD-L1, ctDNA tested pretherapy, ctDNA tested 6 weeks after randomization (post-treatment), ctDNA clearance, tTMB, TCGA-subtype and tGE3 50. The statistical analysis was conducted using chi-square test. (H) Demonstration of the degree of activation of 14 tumor signaling pathways in four subgroups of the immunotherapy cohort in the bar plot. (I-J) From the differentially expressed genes between the H-IE and H-IE groups of immunotherapy cohort, the top 100, 200 and 300 genes were screened out for KEGG (I) and GO (J) enrichment analysis, and some representative pathways were selected with an adjusted *, $p < 0.05$; **, $p < 0.01$; ***, $p < 0.001$; ****, $p < 0.0001$; NS: $p > 0.05$. tTMB, Tumor mutation burden; PD-L1, Programmed cell death-ligand 1; H-IE, High-UAIscore Immune-Enriched; L-IE, Low-UAIscore Immune-Enriched; H-ID, High-UAIscore Immune-Desert; L-ID, Low-UAIscore Immune-Desert.
